# The *Traf2*DNx*BCL2-tg* Mouse Model of Chronic Lymphocytic Leukemia/Small Lymphocytic Lymphoma Recapitulates the Biased IGHV Gene Usage, Stereotypy, and Antigen-Specific HCDR3 Selection of Its Human Counterpart

**DOI:** 10.3389/fimmu.2021.627602

**Published:** 2021-04-12

**Authors:** Gema Perez-Chacon, Juan M. Zapata

**Affiliations:** ^1^ Instituto de Investigaciones Biomédicas “Alberto Sols”, CSIC-UAM, Madrid, Spain; ^2^ Instituto de Investigación Hospital Universitario La Paz (IDIPAZ), Madrid, Spain

**Keywords:** TRAF2, BCL2, chronic lymphocytic leukemia, CLL, small lymphocytic lymphoma, IGHV, BCR stereotipy

## Abstract

Chronic lymphocytic leukemia (CLL)/Small lymphocytic lymphoma (SLL) is a heterogeneous disease consisting of at least two separate subtypes, based on the mutation status of the immunoglobulin heavy chain variable gene (IGHV) sequence. Exposure to antigens seems to play a role in malignant transformation and in the selection and expansion of more aggressive CLL clones. Furthermore, a biased usage of particular IGHV gene subgroups and the existence of stereotyped B-cell receptors (BCRs) are distinctive characteristics of human CLL. We have previously described that *Traf2*DN/*BCL2* double-transgenic (tg, ^+/+^) mice develop CLL/SLL with high incidence with aging. In this model, TNF-Receptor Associated Factor (TRAF)-2 deficiency cooperates with B cell lymphoma (BCL)-2 in promoting CLL/SLL in mice by specifically enforcing marginal zone (MZ) B cell differentiation and rendering B cells independent of BAFF for survival. In this report, we have performed the sequencing of the IGHV-D-J rearrangements of B cell clones from the *Traf2*DN/*BCL2*-tg^+/+^ mice with CLL/SLL. The results indicate that these mice develop oligoclonal and monoclonal B cell expansions. Allotransplantation of the oligoclonal populations into immunodeficient mice resulted in the preferential expansion of one of the parental clones. The analysis of the IGHV sequences indicated that 15% were mutated (M) and 85% unmutated (UM). Furthermore, while the *Traf2*DN/*BCL2*-tg^-/-^ (wild-type), ^-/+^ (*BCL2* single-tg) and ^+/-^ (*Traf2DN*DN single-tg) littermates showed the expression of various IGHV gene subgroups, the CLL/SLL expanded clones from the *Traf2*DN/*BCL2*-tg^+/+^ (double-transgenic) mice showed a more restricted IGHV gene subgroup usage and an overrepresentation of particular IGHV genes. In addition, the HCDR3-encoded protein sequence indicates the existence of stereotyped immunoglobulin (Ig) in the BCRs and strong similarities with BCR recognizing autoantigens and pathogen-associated antigens. Altogether, these results highlight the remarkable similarities between the CLL/SLL developed by the *Traf2*DN/*BCL2*-tg^+/+^ mice and its human counterpart.

## Introduction

Chronic lymphocytic leukemia (CLL) is the most common adult leukemia in the Western world. CLL and small lymphocytic lymphoma (SLL) are two manifestations of the same B cell neoplasia and are characterized by the accumulation of slowly proliferating CD5^+^CD23^+^ B lymphocytes with dysregulated apoptosis (1–3).

It is well established that CLL is a heterogeneous disease consisting of at least two separate subtypes, based on phenotypic and clinical behavior. Approximately 55% of CLL patients have mutated (M) immunoglobulin heavy chain variable (IGHV) genes (4–6), which have a better prognosis than patients with unmutated (UM)- IGHV genes (6–8). According to phenotypic analysis and gene expression profiling both M- and UM-CLL are antigen-experienced B cells (9, 10). The differences in clinical outcome and biological characteristics between CLL patients with M- and UM-IGHV genes could be related to distinct differences in mutation incidence and distribution reflecting specific underlying mutagenic mechanisms between these two groups (11). As a result, M- and UM-CLL show differences in BCR reactivity profile (12) and signaling (13).

In addition, CLL can also be classified according to the expression of stereotyped HCDR3, which are found in a 41% of CLL patients (14, 15). Indeed, the remarkable similarity of HCDR3 regions within sets of patients strongly supports the notion that B cell receptor (BCR) recognition of particular antigens is a driving force in clonal selection, expansion and evolution in CLL [reviewed in (16, 17)].

CLL cells are low proliferating cells, mostly quiescent and with dysregulated apoptosis. Only a small percentage are proliferating cells, which makes difficult their expansion in immunodeficient mice. Besides, human CLL cell xenotransplantation may results in the expansion of B cell clones that do not recapitulate the IGHV-D-J rearrangements of the parental clone [reviewed in (18)]. In addition, it has been shown that donor T cells are required to support CLL implantation (19). However, proliferating T cells could result in a graft *versus* host disease that hampers the utility of CLL xenotransplanted mice.

Mouse models of CLL are useful tools for the study of CLL etiology and as preclinical platforms for new drug testing. Several CLL mouse models are currently available, which recapitulate key aspects of the human disease [reviewed in (20)]. However, a majority of these CLL mouse models, including the profusely studied E*µ-T Cell Leukemia-1* (E*µ-TCL-1*)-tg mice [reviewed in (21)], only produce UM-CLL clones, thus implying that M-CLL etiology is not properly represented in these mice.

We previously described that B cell-specific *Traf2*DN*/BCL2*-double-tg (^+/+^) mice develop CLL/SLL with high incidence (22, 23). In this mouse model, expression of TRAF2DN causes the depletion of endogenous TRAF2, resulting in unbridled BAFF signaling and constitutive NFKB2 activation, causing the expansion of marginal zone (MZ) B cells (24). BCL2 overexpression, which is a CLL trademark (25), would provide MZ B cells with non-redundant and complementary protection against apoptosis that predisposes these cells to CLL/SLL.

In this report we show that the CLL/SLL arising in the *Traf2*DN*/BCL2*-tg^+/+^ mice consists of expanded M- and UM-CLL/SLL clones. Expanded clones show a biased IGHV gene usage, stereotypy and express HCDR3 that are similar to those recognizing autoantigens and pathogen antigens, thus closely resembling human CLL.

## Materials and Methods

### Transgenic Mice

Lymphocyte-specific *Traf2*DN-tg expressing a 1D4-epitope–tagged TRAF2 deletion mutant lacking the N-terminal 240 amino acids (AA) encompassing the RING and zinc finger domains (TRAF2DN) (26) and B cell-specific *BCL2*-tg mice mimicking the t(14;18)(q32;21) translocation involving *BCL2* and *IgH* found in human follicular lymphoma (27) have been previously described. *Traf2*DN-tg (FVB/N) and *BCL2*-tg (BALB/c) heterozygous mice were bred to produce F1 litters with progeny of the four possible genotypes ((wild-type ^-/-^; *Traf2*DN-tg (single-positive, ^+/-^); *BCL2*-tg (single-positive, ^-/+^); and *Traf2*DN*/BCL2* (double-positive, ^+/+^)) expressed on FVB/N x BALB/c mixed background as previously described (22). Analysis of the transgenic mouse genotypes was performed by polymerase chain reaction (PCR) using primers specific for *Traf2* (F) 5’-GACCAGGACAAGATTGAGGC-3’ and (R) 5’-GCACATAGGAATTCTTGGCC-3’) and *BCL2* (F) 5’-TTAGAGAGTTGCTTTACGTGGCCTG-3’ and (R) 5’-ACCTGAGGAGACGGTGACC-3’. The animal protocols were approved by the Bioethics Committee of the hosting institution. Mice showing symptoms of distress and pain (heavy breath, weight loss, distended belly, respiratory distress, lethargy, etc) were euthanized. All transgenic mice in the study were heterozygotes for each transgene.

### Isolation of Mononuclear Cells

Spleens, lymph nodes and blood from *Traf2*DN*/BCL2*-tg mice of the different genotypes were collected and mononuclear cells were isolated by Ficoll density centrifugation (Lympholyte-M; Cedarlane Laboratories, Burlington, NC).

### Flow Cytometry Analysis

Mononuclear cells were incubated with 50 μg/ml human γ-globulin for 10 minutes at 4°C. Then, 10^6^ cells were incubated with a combination of FITC-, PE-, or APC-conjugated antibodies against mouse CD45R/B220, CD21, CD23, IgM, IgD, CD5, and CD43 (all from BD Biosciences). After 30 minutes of incubation at 4°C, cells were washed with PBS and analyzed by flow cytometry in a FACSCanto II cytofluorimeter and the FACSDiVa 6.1.2 (BD Biosciences) flow cytometry analysis software.

### Immunohistochemistry

Tissues and organs from transgenic mice were fixed in 10% formalin (Sigma-Aldrich) or in Bouin’s solution (Sigma-Aldrich) for bone marrow analysis and embedded in paraffin. Tissue sections (5 μm) were deparaffinized and then stained with hematoxylin and eosin, dehydrated, and mounted in DPX (Fluka). Blood smears were stained with Wright-Giemsa (Sigma-Aldrich).

### Immunoglobulin IGHV-D-J Sequence Analysis

Tissues and cells from *Traf2*DN*xBCL2* mice representative of all different genotypic combinations (^-/-^; ^+/-^; ^-/+^ and ^+/+^) were extracted and total RNA was isolated using TRI_ZOL_ reagent and the PureLink™ RNA mini kit (Life Technologies, Carlsbad, CA), following the manufacturer’s instructions. The obtained RNA was reverse transcribed into cDNA using 2 U Superscript II reverse transcriptase (Life Technologies). The IGHV-D-J regions were amplified following a modified protocol (28), using the following primers: IGHV primer (F) 5’-SARGTBMAGCTGSAGSAGTCWGG-3’; CHµ primer (R) 5’-CAGATCTCTGTTTTTGCCTCGTA-3’; CHγ primer (R) 5’-ATGCAAGGCTTACACCACAATCC-3’ and CHα primer (R) 5’-TAATAGGAGGAGGAGGAGTAGGAC-3’ (S: G/C; R: A/G; B: C/G/T; M: A/C; W: A/T). The conditions of the PCR reaction were: one cycle of denaturing at 94°C for 10 minutes, followed by 38 cycles of denaturing at 94°C for 1 minute, annealing at 52°C for 1 minute and extension at 68°C for 1 minute, with a final extension step at 68°C for 10 minutes. The PCR products were then analyzed by gel electrophoresis on a 2% agarose gel, excised and purified (Qiagen). Purified products were cloned using the pGEM^®^-T Vector System (Promega, Madison, WI, USA), following the manufacturer’s instructions. From 5 to 15 colonies of each sample were grown up in culture overnight and the plasmids were extracted using the Wizard^®^ Plus SV Minipreps DNA Purification System (Promega). Miniprep products were sequenced in a capillary sequencer by GATC Biotech (Konstanz, Germany). Nucleotide sequences were analyzed by means of Chromas 2.4.3 software (Technelysium, Queensland, Australia) and compared with those mouse germ line (GL) sequences available in the IMGT repertoire IG database using the IMGT/V-QUEST analysis tool (29). Since our mice are FVB/N x BALB/c F1 hybrids and the GL of these strains are underrepresented (BALB/c) or absent (FVB/N) in the IMGT repertoire IG database, to discriminate between *bona fide* somatic hypermutation (SHM) and strain-specific IGHV gene polymorphism (SSP), a clustal W multiple sequence analysis of the IGHV sequences from the clones with identical IGHV genes (n ≥ 3) found in the *Traf2*DNx*BCL2*-tg and *Traf3*x*BCL2*-tg mice irrespective of their genotype (both FVB/N x BALB/c F1 hybrids) was made. A detailed description of the criteria used to discriminate between SSP and SHM is provided in **Supplementary Materials and Methods**. Sequences ≥ 98% identity to the corresponding GL IGHV gene sequence were considered unmutated (UM). Isolectric point (pI) of HCDR3 region was calculated with the Compute pI/Mw tool (ExPASy Bioinformatics Resource Portal, http://web.expasy.org/compute_pi/). HCDR3 analysis was carried out comparing the sequence in the protein BLAST database.

### Statistics

IBM SPSS statistics v.26 (SPSS, Chicago, IL) and Graph Pad Prism 5 were used for statistical analysis. Statistical significance for HCDR3 length and isoelectric point (pI) was determined using the *t*-Student test. Pearson Chi-Square and likelihood ratio tests with Monte Carlo correction were applied for assessing the significance of the IGHV-D-J subgroups distribution among genotypes. Proportion test was used to determine the significance of IGHV gene expression frequency.

## Results

### Characteristics of BCRs Expressed by CLL/SLL B Cells From the *TRAF2*DNx*BCL2*-tg^+/+^ Mice

**Figure 1:**
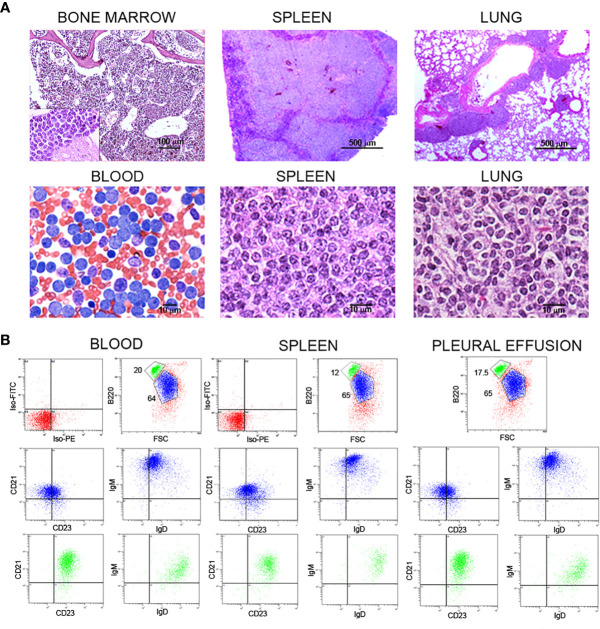
Histochemical and flow cytometry analysis of B cell populations and tissues from representative *Traf2*DNx*BCL2*-tg^+/+^ mice with CLL/SLL. **(A)** Histochemical analysis of bone marrow, spleen, lung and blood from one representative *Traf2*DNx*BCL2*-tg^+/+^ mouse that had developed CLL/SLL is shown. H&E staining was used for bone marrow (x10 and x100), spleen (x4 and x100) and lung (x4 and x100), and Wright-Giemsa staining for the blood smear (x60). Scale bars are shown. **(B)** Three-color flow cytometry analysis was performed to determine the phenotype of expanded B lymphocyte populations. Gating of the expanded population was based on the CD45R/B220 and FSC plot of each sample analyzed and is indicated in the figure. Plots show CD21/CD23 and IgM/IgD expression for the expanded B cell population (FSC^large^/B220^low^, blue) and of the normal B2 population (FSC^small^/B220^high^, green). The quadrants settings were selected based on the staining of isotype controls. The tissue source of the analyzed lymphocytes is indicated in the figure.

As stated above, *Traf2*DNx*BCL2*-tg^+/+^ mice develop CLL/SLL with high incidence as they age (22). In most mice, SLL arises first, involving splenomegaly, lymphadenopathy and infiltration of different tissues and organs, later progressing to CLL (22, 23). An example of the histology features of the bone marrow, blood, spleen and lung of a representative *Traf2*DNx*BCL2*-tg^+/+^ mouse with CLL/SLL is shown in **Figure 1A**. In addition, flow cytometry analysis of the B cell populations in this mouse (**Figure 1B**) identified two B cell populations. One majority population, with larger cells based on their forward scatter (FSC) profile and expressing low levels of B220, IgD, CD21 and CD23 and high levels of IgM (**Figure 1B**), corresponds to the CLL/SLL expanded cells (blue). These cells were CD43^high^ and CD5^low or null^ (not shown). The other population (FSC^small^) is composed by seemingly normal B2 cells expressing B220^high^, IgM^low^, IgD^high^, CD21^high^, and CD23^middle^ (green) (**Figure 1B**). These cells were CD43^null^ and CD5^null^ (not shown). The expanded CLL/SLL population is found in blood, spleen and in pleural effusion (**Figure 1B**).

To ascertain the BCR characteristics of these CLL/SLL cells, we have analyzed the sequences of the HCDR3 of these mice. **Table 1** shows the HCDR3 features and frequency of the expanded clones isolated from *Traf2*DN*xBCL2*-tg^+/+^ mice with CLL/SLL. Based on the HCDR3 sequences, these mice develop oligoclonal (mice: #13, #16, #65, #72 and #74) and monoclonal (mice: #29, #40, #45, #50 and #51) B cell expansions (**Table 1**). Interestingly, when spleen and blood were compared, we have examples of mice with identical expanded clones in both sources (mice: #16, #40 and #65) but also a mouse (#55) with different clones in spleen and blood.

**Table 1:** Characteristics of the expanded CLL/SLL clones from the *Traf2*DNx*BCL2*-tg +/+ mice.

Animal no.	Age (months)	Sex	Tissue	IGHV Family IMGT	IGHV gene IMGT	IGHV gene Vbase2	IGD family IMGT	IGD gene	IGHJ gene	SHM %	SHM status	Frequency	%	HCDR3	HCDR3 lenght	pI
**13**	**13**	**F**	**Spleen**	VH14	IGHV14-2*02 F	VHSM7.a2psi.88	D2	DSP2.9	JH4	1.1	UM	5/13	38	**GRDDGYYYAMDY**	12	3.93
				VH5	IGHV5-17*02 F	VH7183.a47.76	D3	DST4.3	JH4	2.1	M	3/13	23	A**RE**GP**RRD**YYAM**D**Y	14	6.16
**16**	**12**	**M**	**Spleen**	VH1	IGHV1-85*01 F	VHJ558.88.194	D1	DFL16.1	JH3	0.35	UM	6/10	60	ASYAFAY	7	5.57
				VH5	IGHV5-17*02 F	VH7183.a47.76	D2	DSP2.2	JH4	0.35	UM	3/10	30	AS**R**STMIIM**D**Y	11	5.88
			**Blood**	VH5	IGHV5-17*02 F	VH7183.a47.76	D2	DSP2.2	JH4	0.35	UM	7/10	70	AS**R**STMIIM**D**Y	11	5.88
				VH1	IGHV1-85*01 F	VHJ558.88.194	D1	DFL16.1	JH3	0.7	UM	3/10	30	ASYAFAY	7	5.57
**29**	**20**	**F**	**Spleen**	VH1	IGHV1-80*01 F	VHJ558.83.189	D2	DSP2.2	JH4	2.1	M	8/10	80	ASPSY**D**YPYYYAM**D**Y	15	3.56
**40**	**11**	**F**	**Spleen**	VH5	IGHV5-17*02 F	VH7183.a47.76	D2	DSP2.6	JH4	0	UM	8/10	80	ATYYGY**DR**VYYYAM**D**Y	16	4.21
			**Blood**	VH5	IGHV5-17*02 F	VH7183.a47.76	D2	DSP2.6	JH4	0.35	UM	9/10	90	ATYYGY**DR**VYYYAM**D**Y	16	4.21
**45**	**20**	**F**	**Spleen**	VH1	IGHV1-9*01 F	VHJ558.b9	D2	DSP2.2	JH3	0.7	UM	8/10	80	A**R**G**D**Y**D**G**E**FAY	11	4.03
**50**	**18**	**M**	**Spleen**	VH1	IGHV1-74*04 F	V102	D2	DSP2.4	JH4	0.35	UM	10/10	100	**ASGYDYAMDY**	10	3.56
**51**	**18**	**M**	**Spleen**	VH1	IGHV1-9*01 F	VHJ558.b9	D4	DQ52	JH4	0.7	UM	10/10	100	A**R**GNW**D**FYYAM**D**Y	13	4.21
**55 P**	**16**	**F**	**Spleen**	VH1	IGHV1-74*04 F	V102	D2	DSP2.4	JH4	0	UM	10/10	100	**ASGYDYAMDY**	10	3.56
			**Blood**	VH5	IGHV5-17*02 F	VH7183.a47.76	D2	DSP2.9	JH4	0.35	UM	5/10	50	AVYVIY**D**GYYGAM**D**Y	15	3.56
				VH1	IGHV1-77*01 F	VHJ558.80.186	D1	DFL16.1e	JH2	1.4	UM	5/10	50	A**R**GG**D**Y	6	5.88
**55 F1**	**-**	** **	**Node**	VH1	IGHV1-77*01 F	VHJ558.80.186	D1	DFL16.1e	JH2	0.7	UM	9/10	90	A**R**GG**D**Y	6	5.88
**65**	**12**	**M**	**Spleen**	VH5	IGHV5-17*02 F	VH7183.a47.76	D3	DST4.3	JH2	0	UM	2/9	22,2	ALGAGYF**D**Y	9	3.8
				VH1	IGHV1-69*02 F	VH124	D2	DSP2.9	JH1	4.9	M	2/9	22,2	A**R**GN**D**GSYWYF**D**V	13	4.21
				VH3	IGHV3-5*02 F	VH36-60.a5.112	D3	DST4	JH4	1.0	UM	2/9	22,2	A**R**I**R**GGAM**D**Y	10	8.79
			**Blood**	VH14	IGHV14-2*02 P	VHSM7.a2psi.88	D2	DSP2.9	JH4	0.35	UM	3/10	30	**GRDDGYYYAMDY**	12	3.93
				VH5	IGHV5-17*02 F	VH7183.a47.76	D3	DST4.3	JH2	0.7	UM	7/10	70	ALGAGYF**D**Y	9	3.8
**72 P**	**15**	**F**	**Spleen**	VH14	IGHV14-2*02 P	VHSM7.a2psi.88	D2	DSP2.9	JH4	0.7	UM	4/14	29	**GRDDGYYYAMDY**	12	3.93
				VH1	IGHV1S130*01 [F]	Unknown	D2	DSP2.2	JH2	0.35	UM	4/14	29	A**R**V**R**NW**D**F**ED**Y	11	4.56
**72 F1**	**-**	** **	**Spleen**	VH1	IGHV1S130*01 [F]	Unknown	D2	DSP2.2	JH2	0	UM	7/10	70	A**R**V**R**NW**D**F**ED**Y	11	4.56
**74 P**	**15**	**F**	**Spleen**	VH1	IGHV1S130*01 [F]	Unknown	D1	DFL16.1	JH2	0	UM	6/10	60	ASGP**D**F**D**Y	8	3.56
				VH1	IGHV1-9*01 F	VHJ558.b9	D2	DSP2.4	JH4	0	UM	3/10	30	A**R**GGYYGY**D**G**D**YYAM**D**Y	17	3.93
**74 F1**	**-**	** **	**Spleen**	VH1	IGHV1-9*01 F	VHJ558.b9	D2	DSP2.4	JH4	0.7	UM	8/8	100	A**R**GGYYGY**D**G**D**YYAM**D**Y	17	3.93

Table shows the mouse ID number, the tissue source of the mRNA sample, the age and the sex of the mice. The immunoglobulin IGHV, IGHD and IGHJ subgroups and genes found recombined in each CLL B cell clone are indicated, according to IMGT/V-QUEST and Vbase2 analysis tools. SHM status indicates whether the IGHV region is unmutated (UM; ≤ 2% difference from the GL sequence) or mutated (M; >2% difference from the GL sequence) after correcting for SSP as described in **Supplementary Materials and Methods**. The frequency and % of occurrence of the B cell clones isolated from the indicated tissues of each mouse is also shown. All clones encoded a productive Ig and the HCDR3 sequence is also provided. Basic (red) and acid (green) AAs are highlighted and stereotyped HCDR3 are shown in bold. The length and isoelectric point (pI) of the HCDR3 sequence are shown. Additional information pertaining to these CLL clones is provided in **Supplementary Table 4**.

In addition, blood lymphocytes or splenocytes (40-60 x 10^6^) from representative *Traf2*DN*xBCL2*-tg^+/+^ mice (#55, #72 and #74) were allotransplanted into immunodeficient SCID/NOD mice. Animals were euthanized when they develop any sign of illness (distended belly, respiratory distress, lethargy, etc). As shown in **Table 1**, only one of the expanded CLL/SLL clones found in each of the parental mice was selectively expanded in the immunodeficient allotransplanted mice.

### IGHV-D-J Subgroups and Gene Usage by the Expanded CLL/SLL Cells From the *TRAF2*DNx*BCL2*-tg^+/+^ Mice

**Figure 2:**
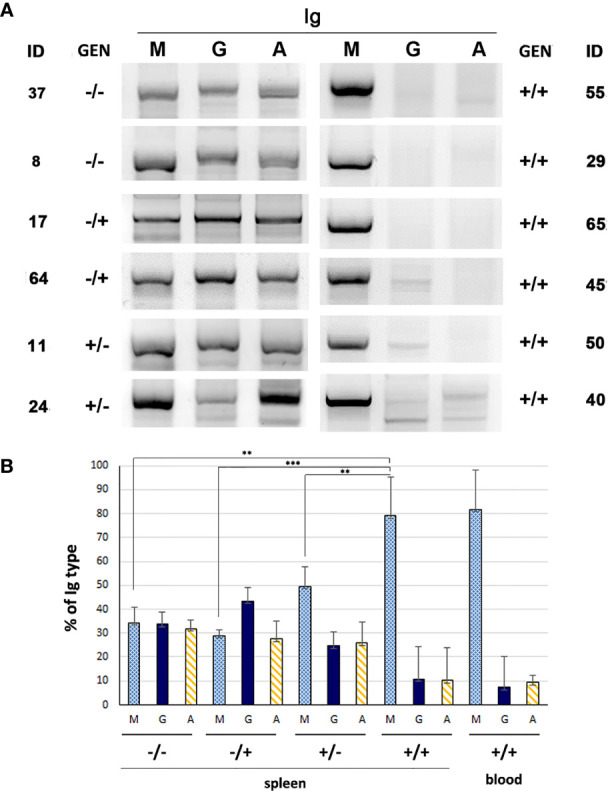
Immunoglobulin subtypes found in *Traf2*DN*xBCL2*-tg mice representative of all different genotypic combinations (^-/-^; ^+/-^; ^-/+^ and ^+/+^). **(A)** mRNA from the indicated mice was extracted and retrotranscribed into cDNA using random primers. Then, PCR was performed using specific primers for the IGHV-D-J region of IgM, IgG or IgA, as described in Materials and Methods. The amplified PCR fragments were analyzed in 2% agarose gels and staining with SYBR Safe and UV light. **(B)** IgM, IgG and IgA cDNAs from mice of the various genotypes (^-/-^, n = 5; ^-/+^, n = 3; ^+/-^, n = 4; ^+/+^, spleen, n = 8, blood, n = 3) were analyzed as indicated in A and the resulting bands were quantified. The results show the percentage of each Ig (M, G and A) found in mice of the various genotypes (average ± SD), Statistical significance: **p < 0.005; ***p < 0.0005).

CLL clones from human CLL patients express mostly IgM and have a biased usage of IGHV genes compared to normal B cells [reviewed in (30)]. These characteristics are also shared by the E*µ-TCL-1*-tg (28, 31) and the *MDR* ^−/−^ and *miR-15a/16-1* ^−/−^ (32) mouse models of CLL. Thus, to ascertain whether B cells from the *Traf2*DNx*BCL2*-tg^+/+^ mice with CLL/SLL have similar characteristics, we have analyzed the Ig isotypes and IGHV-D-J rearrangements expressed by B cells from these mice and compared them with those found in mice representing all other genotype combinations. For this purpose, *Traf2*DN-tg (FVB/N background) and *BCL2*-tg (BALB/c background) mice were crossed to produce F1 litters with mice harboring the different transgene combinations, *Traf2*DN*xBCL2*-tg^-/-^, ^+/-^, ^-/+^ and ^+/+^. The analyses were performed when the *Traf2*DNx*BCL2*-tg^+/+^ mice developed CLL/SLL, using for comparison age- and sex-matched mice representing all genotypes and genders. As shown in **Figure 2A**, retrotranscription and amplification of the mRNAs encoding for IgM, IgG and IgA shows that B cells from *Traf2*DNx*BCL2*-tg^+/+^ mice with CLL/SLL almost exclusively express IgM, while all three Igs (M, G and A) mRNAs could be readily detected in B cells from representative mice of all the other genotypes. The relative expression of IgM, IgG and IgA in the *Traf2*DNx*BCL2*-tg with the different genotypes and in the expanded CLL/SLL clones is shown in **Figure 2B**.

**Figure 3:**
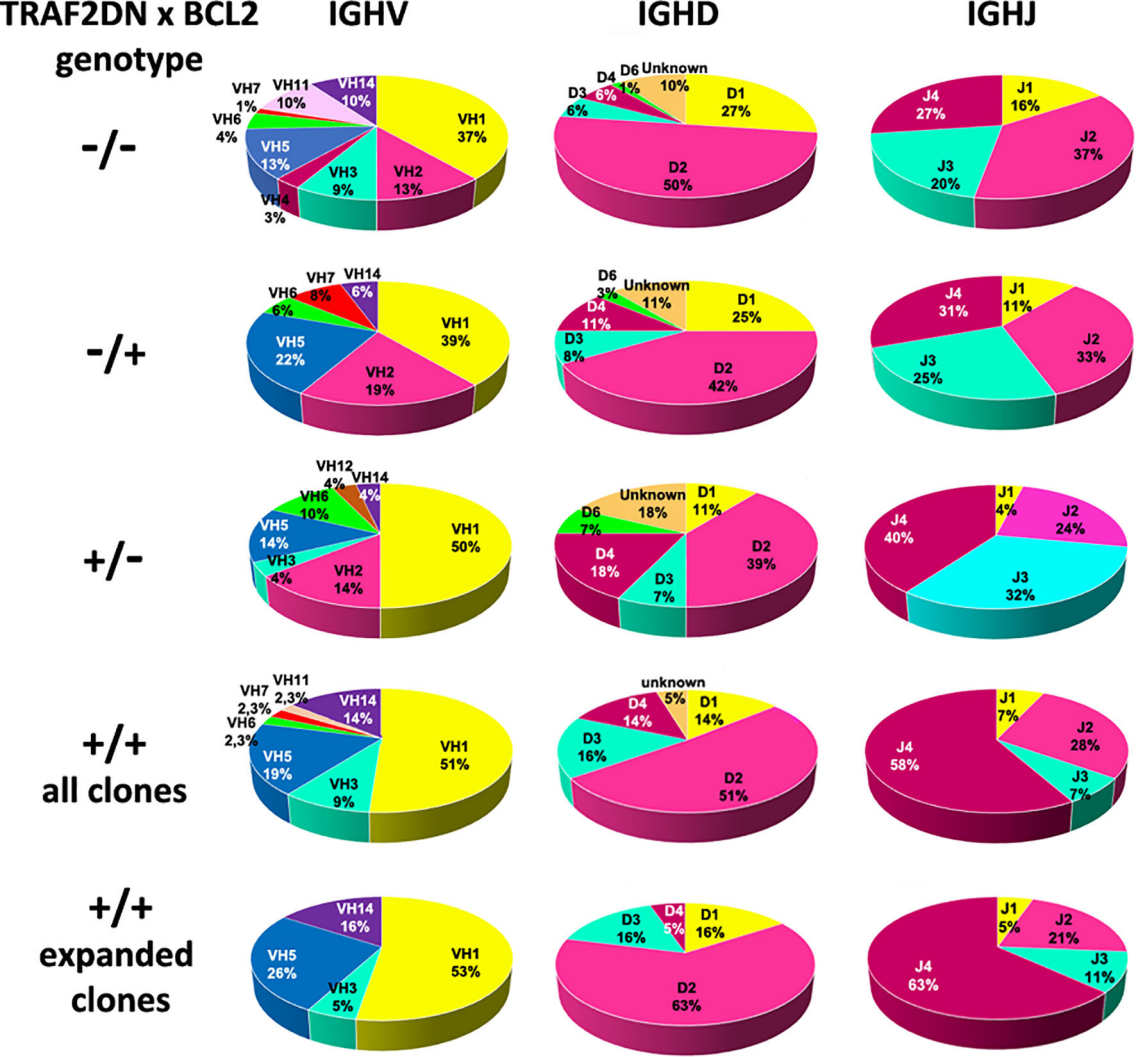
Analysis of the IGHV, IGHD and IGHJ genes subgroup usage in *Traf2*DN*xBCL2*-tg. Circle diagrams representing the percentage of the IGHV, IGHD and IGHJ subgroup usage of mice representative of all different genotypic combinations (^-/-^; ^+/^-; ^-/+^ and ^+/+^), including those found in the *Traf2*DN*xBCL2*-tg^+/+^ expanded CLL/SLL clones are shown.

Next, we studied whether a biased IGHV gene usage was also a feature of the CLL/SLL developed by the *Traf2*DNx*BCL2*-tg^+/+^ mice. The IGHV, IGHD and IGHJ genes and the HCDR3 sequences expressed in the expanded B cell clones from the *Traf2*DN*xBCL2*-tg*^+/+^* mice with CLL/SLL are shown in **Table 1**. In addition, similar information from the *Traf2*DN*xBCL2*-tg mice with ^-/-^, ^+/-^, ^-/+^ genotypes and the whole list of clones isolated from the *Traf2*DN*xBCL2*-tg^+/+^ mice are shown in **Supplementary Tables 1–4**, respectively. A schematic representation of the IGHV, IGHD and IGHJ subgroups expressed in B cells from mice of each genotype and those used by the expanded B cell clones of the *Traf2*DN*xBCL2*-tg^+/+^ mice with CLL/SLL are shown in **Figure 3** and **Supplementary Figure 1**. B cell clones isolated from the *Traf2*DN*xBCL2*-tg^-/-^ (wild-type) mice demonstrated the usage of various IGHV subgroups, with a larger representation of IGHV1 (37%) followed by IGHV2 (13%), IGHV5 (13%), IGHV10 (10%) and IGHV14 (10%) subgroup genes. A similar picture emerges from the analysis of the *Traf2*DN*xBCL2*-tg mice of ^+/-^ and ^-/+^ genotypes, also showing the usage of various IGHV subgroups, with IGHV1 being the most prominently used in all of them, consistent with the larger representation of this subgroup in the murine GL repertoire (33). In contrast, IGHV1 (51%), IGHV5 (19%), IGHV14 (14%) and IGHV3 (9%) are the subgroup genes most conspicuously used by B cells from the *Traf2*DN*xBCL2*-tg^+/+^ mice and also by the expanded CLL/SLL clones (**Figure 3** and **Supplementary Table 5**). Interestingly, the IGHV subgroup expression frequency observed in other CLL mouse models is seemingly different to that of the *Traf2*DN*xBCL2*-tg^+/+^ expanded CLL/SLL clones, with the exception of IGHV1, which is the most expressed IGHV gene subgroup in all of them (**Supplementary Table 5**) (see discussion).

Pearson Chi-square and likelihood ratio (LR) test with the Monte Carlo correction showed that the distribution of IGHV subgroups expressed in the *Traf2*DN*xBCL2*-tg^+/+^ B cell clones compare to those expressed in the ^-/-^, ^+/-^ and ^-/+^ mice was statistically significant (p = 0.069) at 90% confidence, but not at 95% confidence, with a significant LR (0.015).

To determine whether this restricted IGHV gene subgroup usage was a distinctive characteristic of the *Traf2*DN*xBCL2*-tg^+/+^ CLL/SLL model or was instead a general feature that could also be found in the expanded B cell clones of other types of B cell malignancies, we analyzed the IGHV subgroup repertoire used by the mature non-Hodgkin lymphomas (NHL) developed by the *TRAF3*x*BCL2*-tg^+/+^ mice (34). These mice are also F1 hybrids of FBV/N x BALB/c background, and therefore are genetically equivalent to the *Traf2*DN*xBCL2*-tg mice. As shown in **Supplementary Figure 2**, in B cells from the *TRAF3*x*BCL2*-tg^+/+^ mice the IGHV subgroup usage (IGHV1 (43%), IGHV5 (13%), IGHV14 (11.8%), IGHV2 (10.5%) is similar to that found in the wild-type (*Traf2*DN*xBCL2*-tg^-/-^) mice. The expanded B cell clones from the *TRAF3*x*BCL2*-tg^+/+^ mice that have developed post-germinal center (GC) NHL malignancies used more frequently genes from the IGHV1 subgroup genes (56%), similar to the *Traf2*DNx*BCL2*-tg^+/+^ CLL/SLL clones, but the usage of IGHV5 (12.5%) and IGHV14 (6.3%) genes is much reduced compared to the latter. Expression of IGHV2 genes is also found in the *TRAF3*x*BCL2*-tg^+/+^ mice, while it is absent in the *Traf2*DN*xBCL2*-tg^+/+^ mice.

Regarding the usage of the IGHD genes, B cell clones of all genotypes preferentially used IGHD2 subgroup gene members (**Figure 3** and **Supplementary Figure 1**) and no statistical significance was observed in the IGHD subgroup distribution among the various *Traf2*DNx*BCL2* genotypes (p = 0.275; LR = 0.327). In contrast, there is a favored usage of the IGHJ4 gene by the *Traf2*DNx*BCL2*-tg^+/+^ mice (all clones, 58%; expanded CLL clones, 63%) compared to the mice with the other *Traf2*DNx*BCL2* genotype combinations (p = 0.024; LR = 0.024) (**Figure 3**) and also compared to the average IGHJ4 gene usage in mice (21.5%) (35).

Next, we assessed whether *Traf2*DNx*BCL2*-tg^+/+^ CLL/SLL clones show any preferential usage of particular IGHV genes similar to what has been described in human CLL [reviewed in (30)] and the E*µ-TCL-1*-tg mice (28). Indeed, as shown in **Table 2**, we observed that 3 genes are overrepresented in the *Traf2*DNx*BCL2*-tg^+/+^ CLL/SLL clones compared to the B cells from mice of all other genotypes and in the *TRAF3*x*BCL2*-tg^+/+^ mice. Thus, VH7183.a47.76 (IGHV5) is found in 25% of the *Traf2*DNx*BCL2*-tg^+/+^ CLL/SLL clones. It is found recombined to a wide variety of IGHD and IGHJ genes producing distinct HCDR3 sequences (**Table 1**). The expression of this gene is also overrepresented in B cell clones from the *Traf2*DNx*BCL2*-tg^+/+^ (18%) and the *Traf2*DNx*BCL2*-tg^-/+^ (14.3%) mice compared to the 1.86% rearrangement frequency found in the BALB/c strain (33). Proportion test shows that these differences are statistically significant (P>0.0001) (**Table 2**). The expression of these gene in the *TRAF3*x*BCL2*-tg^+/+^ B cell lymphoma clones is not significantly different to the expression in the normal population. Another gene, VHJ558.b9 (IGHV1) is found in 15% of the *Traf2*DNx*BCL2*-tg^+/+^ CLL/SLL clones (13.3% of all *Traf2*DNx*BCL2*-tg^+/+^ B cell clones), a statistically significant difference (P<0.0001) compared to the 1.18% rearrangement frequency found in the C57BL/6 strain (33). It is also overrepresented in the *TRAF3*x*BCL2*-tg^+/+^ B cell clones but not in the expanded B cell lymphoma clones. Finally, VHSM7.a2psi.88 (IGHV14) is rarely or not found in BALB/c and C57BL/6 strains (33), but it was found in 15% of the expanded CLL/SLL clones and in 8.9% of all *Traf2*DNx*BCL2*-tg^+/+^ B cell clones (P<0.0001) (**Table 2**). Interestingly, this VHSM7.a2psi.88 was found recombined to DSP2.9 and IGHJ4 genes in expanded clones from 3 different *Traf2*DNx*BCL2*-tg^+/+^ mice with CLL/SLL, producing an identical HCDR3 sequence (see below).

**Table 2:** A restricted set of IGHV genes predominates in the expanded *Traf2*DN*xBCL2*-tg^+/+^ CLL/SLL clones.

Mouse tg-line	Genotype	IGVH genes (% usage)					
		IGHV5	*P*	IGHV1	*P*	IGHV14	*P*
		VH7183.a47.76		VH558.b9		VHSM7.a2psi88	
***Traf2*DNx*BCL2***	**-/-**	**4.3**	0.12	**1.4**	0.86	**nf**	0.9
	**+/-**	**4**	0.48	**4**	0.004	**nf**	0.87
	**-/+**	**14.3**	**<0.0001**	**6**	0.014	**nf**	0.85
	**+/+**	**18**	**<0.0001**	**13**	**<0.0001**	**2**	**<0.0001**
	+/+ *	**25**	**<0.0001**	**15**	**<0.0001**	**15**	**<0.0001**
***TRAF3*x*BCL*2**	**+/+**	**2.5**	0.5	**8.1**	**<0.0001**	**nf**	0.69
	+/+ *	**6.2**	0.18	**nf**	0.66	**nf**	0.9
**% in mouse (Ref 33)**	**wt**	**1.9**	**-**	**1.2**	**-**	**<0.1**	**-**

The IGHV subgroup, gene and its frequency (%) in the B cell clones isolated from the Traf2DNxBCL2-tg and TRAF3xBCL2-tg mice of the indicated genotypes, as well as the expression frequency (%) of these genes in mice (33) is shown. Statistical significance was calculated using proportion test and significant results are highlighted in bold. Clones from different tissues of the same mouse are only counted once. Clones found in parental and F1 mice are only counted once. +/+* indicated the expanded B cell clones (CLL/SLL in the Traf2DNxBCL2-tg^+/+^ mice and mature non-Hodgkin lymphoma in the TRAF3xBCL2-tg^+/+^ mice). nf, not found.

Altogether, these results suggest that a preferential usage of IGHV subgroups and genes by the expanded CLL/SLL clones from the *Traf2*DNx*BCL2*-tg^+/+^ mice is occurring, similar to what has been previously observed in human CLL patients and in the E*µ-TCL-1*-tg mouse model of CLL.

### Analysis of the IGHV Somatic Hypermutation Status and HCDR3 Features of the CLL/SLL Clones From the *Traf2*DNx*BCL2*-tg^+/+^ Mice

Patients with CLL segregate into two groups based on the number of SHMs in the rearranged IGHV genes of the transformed clones. Approximately 55% of CLL patients have transformed B cells with mutations in IGHV genes (M) (4–6). The rest of the patients have UM IGHV CLL clones, which correlates with poor disease prognosis (6–8).

To determine the frequency of M *vs.* UM IGHV regions in the expanded CLL/SLL clones of the *Traf2*DNx*BCL2*-tg^+/+^ mice we first compared the IGHV sequence of the transformed clones with the available GL sequences stored in the IMGT repertoire IG database [mostly based on the C57BL/6, with scattered presence of 129/sv and BALB/c lines GL sequences (33)]. The results obtained using the IMGT/V-QUEST analysis tool showed that many sequences have considerable variations with their respective GL IGHV genes. A similar result was obtained when the IGHV sequences from the B cell clones isolated from mice with the other genotypes (^-/-^, ^+/-^, ^-/+^ and all ^+/+^) was compared. Since our mice are FVB/N x BALB/c hybrids and because it has been shown that the IGHV GL repertoire and sequence is highly variable among inbred mouse strains (33, 36), these variations might reflect the reported differences among strains and the absence of IGHV GL sequences from the FVB/N mouse line in the IMGT repertoire IG database.

To determine whether some of these variations with the GL sequences may be the result of SSPs, we have performed a clustal W sequence comparison of the IGHV region of *Traf2*DNx*BCL2*-tg B cell clones with identical IGHV alleles, irrespective of their genotype. Indeed, we have observed the existence of nucleotide mismatches compared to the IMGT referenced IGHV gene that are conserved in most of the corresponding IGHV gene from different *Traf2*DNx*BCL2*-tg individuals and genotypes. Since SHM randomly introduces any of the 4 nucleotides in a given spot, a mismatch of the same nucleotide in the same position of several identical alleles compared to the GL sequence strongly suggest the existence of a polymorphism. The number of SSPs found in the IGHV rearranged sequences from the *Traf2*DNx*BCL2*-tg B cell clones ranges from 1 (0.35% of the IGHV sequence) to 24 (9.375%), averaging a 2.54% SSPs, consistent with previously reported IGHV GL differences among mouse strains (36). The criteria for discriminating between SSP and SHM and examples of the IGHV gene sequence comparisons are shown in supplementary materials and methods and **Supplementary Figure 3**, respectively.

The estimation of SHM events (%) according to the criteria described above found in the expanded CLL/SLL B cell clones is shown in **Table 1**. In addition, the percentage of similarity of the IGHV region of the analyzed B cell clones with the GL and the SPP and SHM estimated events for the B cell clones from all the *Traf2*DNx*BCL2*-tg genotypes, is shown in **Supplementary Tables 1–4**. A standard 2% difference with the GL was applied to categorize UM or M IGHV clones (4, 28, 37). As shown in **Supplementary Table 6**, the CLL/SLL expanded clones from the *Traf2*DNx*BCL2*-tg^+/+^ mice were 85% UM and 15% M (identical clones found in a different tissue of the same mouse as well as identical clones found in parental and allotransplanted F1 mice were only counted once). Similar UM and M percentages were found in *Traf2*DNx*BCL2*-tg^+/-^ (78.6% UM *v*s. 21.4% M), and *Traf2*DNx*BCL2*-tg^+/+^ (all clones) (80% UM *vs.* 20% M) mice and a larger population of UM B cell clones was also found in *Traf2*DNx*BCL2*-tg^-/+^ (60% UM *vs.* 40% M). In contrast, *Traf2*DNx*BCL2*-tg^-/-^ B cell clones are split in half (46% UM *vs.* 54% M). This result is consistent with the fact that *Traf2-*deficiency causes the expansion of MZ B cells (24, 38), which are mostly UM (39). In addition, *Bcl2* overexpression has been shown to reduce the SHM rate (40).

**Figure 4:**
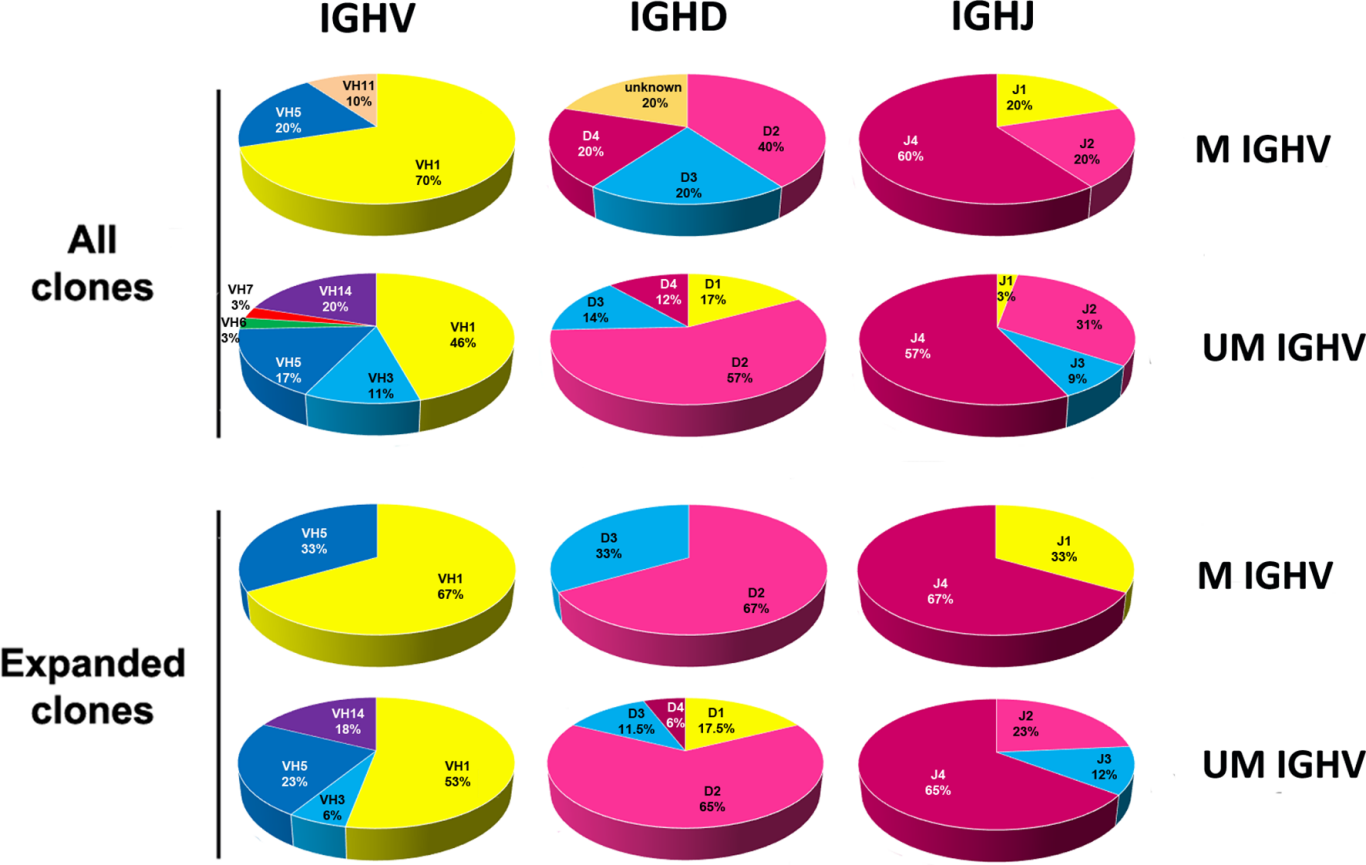
Analysis of the IGHV, IGHD and IGHJ genes subgroup usage in the CLL/SLL clones from the *Traf2*DN*xBCL2*-tg^+/+^ mice according to the SHM status (UM and M). The percentage of usage of the various IGHV, IGHD and IGHJ subgroups found in B cell clones isolated from the *Traf2*DN*xBCL2*-tg^+/+^ mice (all) and those found in the expanded CLL/SLL clones, distributed according to the SHM status (UM and M), are indicated and represented in circle diagrams. A given IGHV gene was considered UM if there was ≤ 2% variation from the GL once corrected for the presence of SSPs.

It has been reported in human CLL patients that UM- and M-CLL clones have a biased usage of IGHV subgroups. Thus, IGHV1 genes predominate in the rearrangements of UM-CLL cells while IGHV3 and IGHV4 genes are more frequently found in M-CLL cells (5, 6, 41). A larger percentage of IGHV1 genes are also found in UM-CLL clones from the E*µ-TCL-1*-tg mice (28) and the *MDR* ^-/-^ and *miR15a/16-1* ^-/-^ mice (32). A comparison between the IGHV, IGHD and IGHJ subgroup usage between M- and UM-clones from the *Traf2*DNx*BCL2*-tg^+/+^ is shown in **Figure 4**. Our analyses showed that UM-B cell clones from the *Traf2*DNx*BCL2*-tg^+/+^ used more frequently IGHV1 (46%), IGHV14 (20%), IGHV5 (17%) and IGHV3 (11%), while M-B cell clones used IGHV1 (70%), IGHV5 (20%) and IGHV11 (10%). A similar trend was observed in UM- and M-CLL/SLL clones, although the reduced number of M-CLL/SLL clones (n=3) avoid any conclusion. In addition, IGHD2 and IGHJ4 genes were overrepresented in both UM- and M-*Traf2*DNx*BCL2*-tg^+/+^ B cell clones and in expanded CLL/SLL clones (**Figure 4**).

Other HCDR3 features, such as length, charge and AA sequence are intrinsic HCDR3 characteristics. HCDR3 sequences vary in their AA composition, charge and length depending on how the IGHV, IGHD and IGHJ genes recombine (5, 42, 43). A summary of the analysis of these HCDR3 features in the expanded *Traf2*DNx*BCL2*-tg^+/+^ B cell clones and of B cells from the other genotypes is shown in **Supplementary Table 6**. The analysis of the HCDR3 AA sequence of the expanded *Traf2*DNx*BCL2*-tg^+/+^ CLL/SLL clones shows an average length of 11.6 ± 2.9 AAs, which is similar to the HCDR3 length in all other genotypes (^-/-^, 11.92 ± 2.7; ^+/-^, 10.71 ± 2.6; ^-/+^, 12.11 ± 3.3; ^+/+^, 11.44 ± 2.8) and in accordance with the HCDR3 average length in mice (11.5 ± 1.9 AAs) (44). Moreover, no remarkable differences were observed between the HCDR3 average length of M *vs.* UM clones in any of the genotypes, including the expanded CLL/SLL clones (**Supplementary Table 6**). Although long HCDR3 have been proposed to be a characteristic of UM-CLL HCDR3 in humans (5, 42), long HCDR3 were found in both M- and UM-clones from mice of the different genotypes, including the expanded CLL/SLL clones. It is noteworthy that the HCDR3 average length of the *Traf2*DNx*BCL2*-tg^+/+^ UM-CLL/SLL clones (11.18 ± 2.9 AAs) is similar to the HCDR3 average length of the UM-CLL clones from the E*µ-TCL-1*-tg mice [11.6 ± 2.3 AAs (28) and 10.6 ± 2.4 AAs (31)] and from the *IgH*-*TEµ* mice [11.4 ± 2.32 (45)]. The average length of the UM-CLL/SLL clones developed by the *MDR* ^-/-^ and *miR15a/16-1* ^-/-^ is slightly longer [12.87 ± 2.17 AAs (32)], but compared to that of the *Traf2*DNx*BCL2*-tg^+/+^ UM-CLL/SLL clones these differences did not reach statistical significance (*P* = 0.073).

The average isoelectric point (pI) of the HCDR3 expressed in the analyzed B cell clones from the *Traf2*DNx*BCL2*-tg mice of the distinct genotypes is also shown in **Supplementary Table 6**. The *Traf2*DNx*BCL2*-tg^+/+^ expanded CLL/SLL clones have the most acidic HCDR3 (4.54 ± 1.31) compared to that of *Traf2*DNx*BCL2*-tg mice with the other genotypes. Indeed, only one *Traf2*DNx*BCL2*-tg^+/+^ CLL/SLL clone had a HCDR3 with a basic pI. Aspartic acid and arginine are the most frequently found acidic and basic AAs, respectively. In addition, tyrosine is frequently overrepresented, with some HCDR3 containing as much as 44% of tyrosine, compared to the average 25% frequency for this AA found in the mouse HCDR3 (44). Of note is that the average pI of the HCDR3 of UM-CLL/SLL clones from the *Traf2*DNx*BCL2*-tg^+/+^ is significantly more acidic than the average pI of the HCDR3 from the E*µ-TCL-1*-tg UM-CLL clones (4.5 ± 1.4 *vs*. 5.9 ± 1.9) (*P*=0.02).

### Identical HCDR3 Are Expressed in *Traf2*DNx*BCL2*-tg^+/+^ CLL/SLL Clones From Distinct Mice

A distinctive characteristic of human CLL is the expression of structurally identical or highly similar HCDR3 between unrelated individuals producing structurally similar BCRs (14, 15, 46). This occurrence is known as HCDR3 stereotypy and points out toward the role of antigens in the clonal selection and pathogenesis of the disease [reviewed in (17, 30, 47)]. Stereotyped HCDR3 rearrangements account for 41% of human CLL clones (15). BCR stereotypes are also found in CLL clones from the E*µ-TCL-1*-tg (28, 31) and from the *MDR* ^−/−^ and *miR-15a/16-1* ^−/−^ (32) mice, among others.

Our results also demonstrate the existence of two identical HCDR3 sequences in the *Traf2*DNx*BCL2*-tg^+/+^ expanded CLL/SLL clones. One is found in mice #50 and #55 (ASGYDYAMDY) and the other in mice #13, #65 and #72 (GRDDGYYYAMDY) (**Table 1**), accounting for the 25% of the CLL/SLL clones. These *Traf2*DNx*BCL2*-tg^+/+^ CLL/SLL stereotyped HCDR3 sequences are found in UM clones, in agreement with the findings in stereotyped HCDR3 sequences from human CLL (17) and from the above mentioned CLL mouse models (28, 32), that are also found in UM-CLL clones. In addition, it is worth noting that other stereotyped sequences are also found in seemingly not expanded clones from the *Traf2*DNx*BCL2*-tg^+/+^ mice (**Supplementary Table 7**). These stereotyped sequences might belong to low represented CLL/SLL clones. Including these low represented clones, we found 18 stereotyped HCDR3 account (31% of all *Traf2*DNx*BCL2*-tg^+/+^ B cell clones), of which 16 of them are UM and 2 are M.

Interestingly, stereotyped HCDR3 sequences were also found in a few B cell clones from the *Traf2*DNx*BCL2*-tg^-/-^, ^+/-^ and ^-/+^ mice (**Supplementary Table 7**). Of note is that some of these stereotyped sequences are found in clones expressing distinct IGHV genes, although always from the same IGHV subgroup. In addition, **Supplementary Figure 4** shows a Clustal W analysis of the IGHV regions from representative clones producing identical HCDR3. Even though these IGHV regions are UM, they differ in their SHM pattern.

It is also worth mentioning that HCDR3 expressed in CLL clones from the E*µ-TCL-1*-tg (28), the *MDR* ^−/−^ and *miR-15a/16-1* ^−/−^ (32) and the *IgH*-*TEµ* (45) mice were also found in *Traf2*DNx*BCL2*-tg^-/-,+/-,+/+^ and in *TRAF3*x*BCL2*-tg^+/+^ mice, although none of these were expanded clones (**Supplementary Table 8**).This result is consistent with the existence of CLL-biased stereotyped BCR in healthy individuals (48, 49).

### Putative Antigens Recognized by the HCDR3 Sequences of the CLL/SLL Clones From the *Traf2*DNx*BCL2*-tg^+/+^ by Comparison With Those in Public Databases

As stated above, CLL cells frequently express BCR recognizing autoantigens and pathogen-associated antigens [reviewed in (16, 17)] that are involved in the clonal selection and progression of the disease (31, 50–54).

The comparison of the BCR HCDR3 sequences of the expanded *Traf2*DNx*Bcl2*-tg^+/+^ CLL/SLL clones with similar sequences found in public databases showed high homology with HCDR3 recognizing autoantigens, such as phosphatidylcholine (82 and 75% homology), cardiolipin (86% homology), dsDNA (80% homology), as well as to pathogen antigens, such as hepatitis C virus E2 protein (81% homology), CMV glycoprotein B (76% homology), *Bordetella* (75% homology) and Vaccinia protein A3 (80% homology) (**Table 3** and **Supplementary Table 9**). Many of these antigens were already described as being recognized by the BCR of human and mouse CLL clones, including phosphatidylcholine (28), cardiolipin (55, 56), dsDNA (55, 57) and CMV (58, 59), further supporting the role of these types of antigens in the etiology of *Traf2*DNx*Bcl2*-tg^+/+^ CLL/SLL.

**Table 3:** HCDR3 sequence alignments of the *Traf2*DNx*BCL2*-tg+/+ CLL/SLL clones and their putative target antigens.

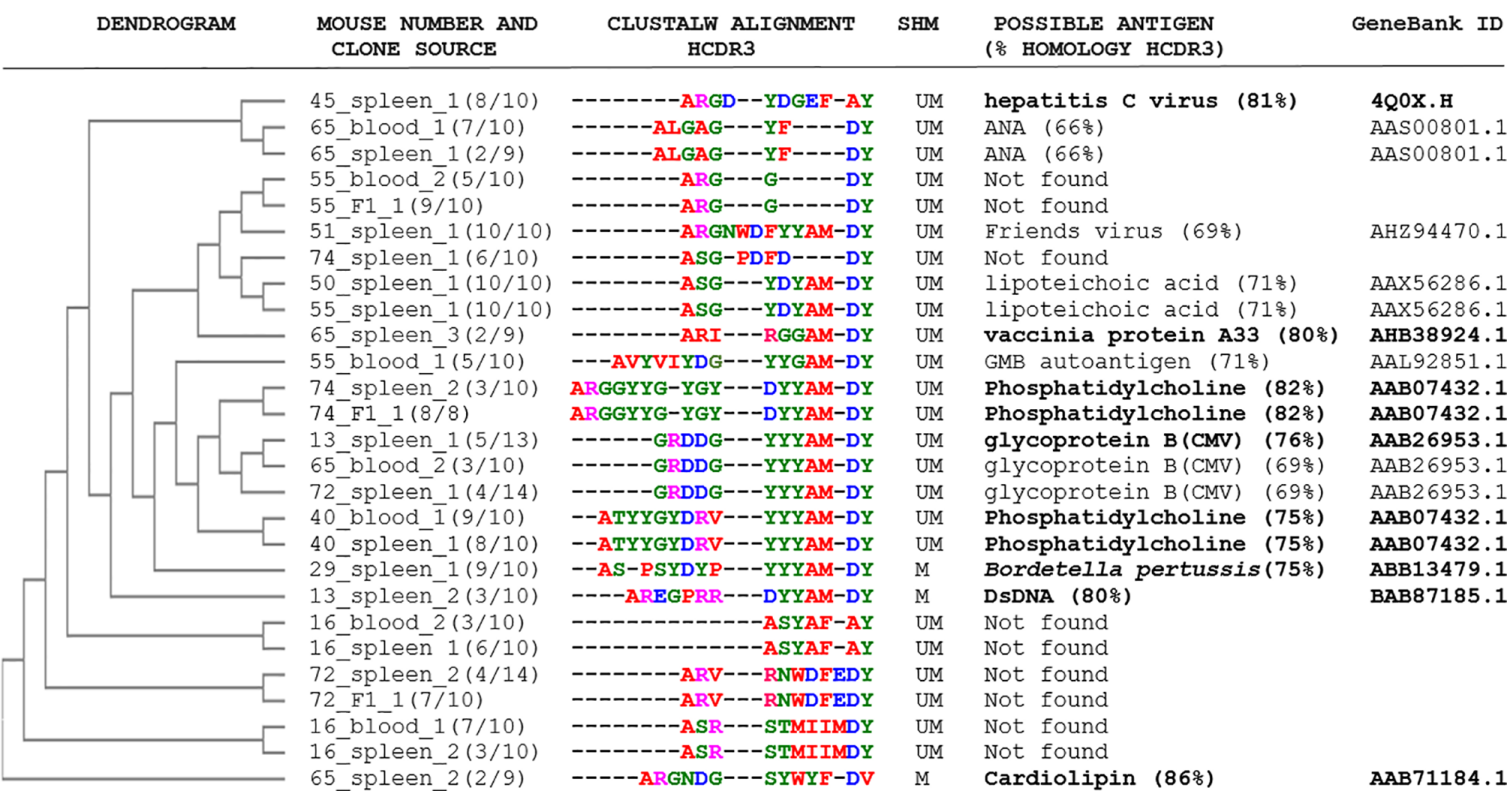

A clustal W alignment and dendrogram comparing the HCDR3 from the expanded Traf2DNxBCL2-tg+/+ CLL/SLL clones is shown. The ID number of the mouse, the source of the tissue, the frequency of occurrence for each clone and the SHM status of the clone (M or UM) are indicated. The putative antigens recognized by the CLL/SLL HCDR3 were determined using NCBI protein Blast (non-redundant sequences restricted to Mus musculus, taxid. 10090) and selecting the antigen recognized by antibodies encoding HCDR3 with the highest similarities to the HCDR3 expressed by the CLL/SLL clones (the antigen candidate and the % of HCDR3 similarities is indicated). Those HCDR3 with ≥ 75% similarities are highlighted. The GenBank accession code for the referred antibodies is provided. Alignment was performed using the clustal W muscle tree method UPGM https://www.ebi.ac.uk/Tools/phylogeny/simple_phylogeny/.

## Discussion

### Characteristics of the HCDR3 of the *Traf2*DNx*BCL2*-tg Mice

The results presented herein underscore the similarities between the CLL/SLL developed by the *Traf2*DNx*BCL2*-tg^+/+^ mice and the CLL developed by human patients. This includes a biased usage of IGHV genes, the existence of CLL/SLL clones with stereotyped HCDR3 and the expansion of CLL/SLL clones with HCDR3 similar to those recognizing autoantigens and bacteria antigenic determinants (1, 30, 60, 61). Furthermore, compared to other CLL mouse models, such as the E*µ-TCL-1*-tg (28) and the *MDR* ^−/−^ and the *miR-15a/16-1* ^−/−^ (32) that only generate UM-CLL clones, the CLL/SLL developed by the *Traf2*DNx*BCL2*-tg^+/+^ mice produce both UM- and M-CLL/SLL clones, similar to human CLL, albeit a vast majority of them are UM.

In this report we have compared the IGHV, IGHD and IGHJ gene usage and the HCDR3 sequences expressed in B cells from mice representing all the different genotypes obtained by crossing heterozygous *Traf2*DN-tg and *BCL2*-tg mice, that is, *Traf2*DNx*BCL2*-tg^−/−^ (wild-type), ^+/−^ (expressing only TRAF2DN), ^−/+^ (expressing only BCL2) and ^+/+^ (expressing both TRAF2DN and BCL2). Our results further demonstrate that monoclonal and oligoclonal B expansions are only observed in the *Traf2*DNx*BCL2*-tg^+/+^ mice that developed CLL/SLL, thus confirming that the expression of both transgenes is necessary to trigger CLL/SLL in these mice (22). In addition, the comparison of B cell clones isolated from *Traf2*DNx*BCL2*-tg mice with all possible transgene combinations reveal a more restricted set of IGHV subgroup and IGHV gene usage by the expanded *Traf2*DNx*BCL2*-tg^+/+^ CLL/SLL clones compared to B cells from mice of the other genotypes. In all genotypes, IGHV1 genes are the most frequently used by B cells from all the different genotypes, in accordance with the fact that IGHV1 is the gene subgroup most prominently used in mice (33). Of note is the lack of IGHV2 genes found in *Traf2*DNx*BCL2*-tg^+/+^ B cells, while this subgroup is readily represented in B cells from mice with the other genotypes and is also found in lymphoma B cell clones from the *TRAF3*x*BCL2*-tg^+/+^ mice. In this regard, it is worth noting that *Traf2*DNx*BCL2*-tg^+/+^ CLL/SLL clones have a MZ origin (24) and that the IGHV2 gene subgroup is underrepresented in transformed B cells of a MZ origin (62, 63)

As described above, the analysis of the IGHV subgroup usage of the expanded *Traf2*DNx*BCL2*-tg^+/+^ CLL/SLL clones indicates a preponderance of certain gene subgroups (IGHV1 > IGHV5 > IGHV14 > IGHV3) (**Supplementary Table 5**). In contrast, the expression of IGHV subgroups found in other CLL mouse models described in the literature is seemingly different. This includes the E*µ-TCL-1*-tg [IGHV1 > IGHV11 = IGHV12 > IGHV4, calculated from (28)], the *MDR* ^−/−^ and *miR-15a/16-1* ^−/−^ [IGHV1 > IGHV11 > IGHV12, calculated from (32)] and the *IgH*-*TEµ*-tg [IGHV1 = IGHV11, calculated from (45)] mice (**Supplementary Table 5**). Although IGHV1 is most frequently found in the CLL clones from all these mouse models, the preferential use of IGHV5 and IGHV14 by the *Traf2*DNx*BCL2*-tg^+/+^ CLL/SLL clones instead of the use of IGHV11 and IGHV12 seen in the other CLL mouse models might indicate that the CLL/SLL developed by the *Traf2*DNx*BCL2*-tg^+/+^ mice arises from a B cell subset different to that of the other CLL mouse models (see below). These differences might also underlie the reported differences in IGHV-D-J usage by mice of different strains (33, 36). In this regard, differences in IGHV subgroup usage have been also observed in CLL from distinct human populations [(64, 65) and references therein]. However, it is noteworthy that mouse IGHV5 and IGHV11 belong to the IGHV clan whose human counterpart is IGHV3, mouse IGHV1 and IGHV14 share clan with human IGHV1, and mouse IGHV3 and IGHV12 are in the same clan than human IGHV4 (66). Interestingly, IGHV3, IGHV1 and IGHV4 are the subgroups most frequently represented in human CLL [(64) and references therein], further stressing the similarities between these CLL mouse models and the human disease. However, it is worth noting human IGHV3, 1 and 4 subgroups contain the larger number of genes and also dominate the repertoire in other physiological and pathological contexts.

Although some mouse CLL clones may have longer HCDR3 than normal B cells, as it has been shown in sets of UM-CLL in humans (5, 42), a comparison of the HCDR3 average length of the *Traf2*DNx*BCL2*-tg^+/+^ UM-CLL/SLL clones and those from the E*µ-TCL-1*-tg, the *MDR* ^−/−^, the *miR-15a/16-1* ^−/−^ and the *IgH*-*TEµ*-tg mice showed no significant differences among them and, in all cases, it was similar to the HCDR3 average length of normal mouse B cells. However, even though this result suggests that this feature of human UM-CLL is not shared by its mouse counterparts, an analysis of a large cohort of CLL samples from 2662 patients have shown that the stereotyped HCDR3 sequences seem to cluster in discrete groups of 9, 13, 20 and 22 AAs (14), thus suggesting that long HCDR3 are not a general feature of human UM-CLL but rather of some stereotype subtypes. Thus, due to the limited number of mouse CLL HCDR3 sequences available, it remains an open question whether a similar distribution could be observed in mouse CLL.

It is important to note that the *Traf2*DNx*BCL2*-tg mice are in a FVB/NxBALB/c mixed background. This is relevant considering that the vast majority of the IGHV sequences available at the IMGT repertoire IG database are from C57BL/6 mice and that IGHV from FVB/N mice are not represented in this database. As stated above, there is a large sequence GL IGHV variation between mouse strains (33, 36). Therefore, a direct comparison of the IGHV sequences from the *Traf2*DNx*BCL2*-tg mice with those GL stored at IMGT would not be representative of the actual percentage of variation of the analyzed IGHV sequences with the GL. To provide a more precise analysis, we have compared all available sequences of the same IGHV gene from the *Traf2*DNx*BCL2*-tg mice (irrespective of their genotype) and *TRAF3*x*BCL2*-tg mice (also in a FVB/NxBALB/c mixed background), as well as with available FVB/N IGHV sequences available in public databases. These comparisons allowed discriminating what differences with the GL sequence were more likely SSP or SHM. Our results indicate that a majority of the IGHV sequences of the *Traf2*DNx*BCL2*-tg -/+; +/- and ++ have ≤ 2% differences with the GL and are categorized as UM. However, it is important to state that these results are an estimation of SHM events and that a comparison with the GL of the mouse strain analyzed is required for an accurate assessment of SHM events.

### Insights Into the Cellular Origins on Mouse CLL

CLL ontogeny is still a matter of intense study and discussion (2, 67, 68). This also applies to the identification of the cellular source of mouse CLL, notwithstanding our deeper knowledge on mouse B cell ontogeny and differentiation compared to that of humans. Questions still remain even on whether human and mouse CLL arises from a single or multiple cell types.

MZ B cells are IgM^+^ cells responding to T-independent antigens. They have a limited IGHV-D-J repertoire usage often producing polyreactive BCR recognizing autoantigens and pathogen antigens (69). MZ B cells are mostly UM but they can go through extra-germinal center SHMs producing also M-MZ B cells (70). Our studies on the mechanisms causing CLL/SLL development in the *Traf2*DNx*BCL2*-tg^+/+^ mice showed that B cell-specific TRAF2DN expression caused proteasome-dependent degradation of endogenous TRAF2, thus rendering B cell-specific *Traf2*DN-tg mice into *bona fide* B cell-specific *Traf2*-deficient mice (24). Confirming previous results (38, 71), we showed that the lack of functional TRAF2 enforces MZ B cell accumulation and releases B cells from the need of BAFF for survival (24). BCL2 overexpression, a defining characteristic of human CLL cells (25) would provide in this model a necessary additional level of protection against apoptosis, likely through a similar mechanism to that described in human CLL (72). Altogether, our results would be consistent with a role for *Traf2*-deficiency and BCL2 overexpression in promoting MZ B cells expansion and predisposing MZ B cells to CLL/SLL transformation (24).

A role for MZ B cells as the source of the CLL/SLL arising in the *Traf2*DNx*BCL2*-tg^+/+^ mice might explain why in this mouse model SLL arises first, later progressing to CLL (22). This would be in line with the ability of MZ B cells to move into circulation (73). Moreover, since the CLL/SLL developed by the *Traf2*DNx*BCL2*-tg^+/+^ mice may express or not CD5 on their surface (22), this could reflect that MZ B cells were at different activation stage at the time of transformation [reviewed in (2)].

On the other hand, various lines of evidence suggest that the CLL developed by other mouse models might arise from a different B cell type. In this regard, there is evidence pointing out to a B1a cell origin for UM-CLL developed by some of the available CLL mouse models [reviewed in (2)]. First, the preferential usage of IGHV1 and IGHV11 genes by the E*µ-TCL-1*-tg (28, 51, 74) and by the *MDR* ^−/−^ and *miR-15a/16-1* ^−/−^ mice (32) is similar to the preferential IGHV subgroup usage of mouse splenic B1a cells (75). Second, the most frequently expressed clones in B1a cells (both, peritoneal and splenic) have HCDR3 with the sequences MRYGNYWYFDV, MRYSNYWYFDV, MRYGSYWYFDV and MRYGSSYWYFDV (75) found in BCRs that are reactive to phosphatidylcholine (28). These HCDR3 are commonly found in expanded CLL clones from the E*µ-TCL-1*-tg mice (28) the *MDR* ^−/−^ and *miR-15a/16-1* ^−/−^ mice (32) and the *IgH*-*TEµ* mice (45) (**Supplementary Table 8**). Third, Hayakawa and coworkers (52) have shown that allotransplantation of B1 cells, but not of other B cell subtypes, from the Eμ-*TCL1*-tg mice resulted in CLL with a biased repertoire, including stereotyped BCRs, thus recapitulating the CLL developed by the Eμ-*TCL1*-tg mice.

Of note is that both MZ B cells and B1 cells have been proposed as a possible source for CLL cells [reviewed in (2)]. However, there is conflicting evidence for MZ B cells as the source of human CLL (2, 67) and we are still lacking clear evidence on the existence of a human counterpart of mouse B1 cells. Therefore, despite the high similarities of the CLL developed by humans and the available mouse CLL models, including the *Traf2*DNx*BCL2*-tg^+/+^ mice, additional research is needed to elucidate whether mouse and human CLL have a similar ontogeny and cell type origin.

### Possible Role of Autoantigens and Pathogen Antigens in the CLL/SLL Developed by the *Traf2*DNx*BCL2*-tg^+/+^ Mice

Although CLL cells relying on antigen-independent, cell-autonomous BCR signaling have been described (76), there is ample evidence for the role of autoantigen-stimulated BCR in CLL clonal selection, expansion and clonal evolution (31, 50–54). Our results showing the similarities of the HCDR3 expressed by the expanded *Traf2*DNx*BCL2*-tg^+/+^ CLL/SLL clones to those recognizing autoantigens and pathogens suggest that antigen-stimulation would also drive disease progression in our CLL/SLL mouse model, similarly to what has been demonstrated in the E*µ-TCL-1*-tg mice (31, 51).

Stereotyped HCDR3 sequences are mostly found in UM-CLL clones in humans and produce BCRs that frequently recognized autoantigens [reviewed in (16, 17)]. In agreement with these findings, the identical HCDR3 found in the *Traf2*DNx*BCL2*-tg^+/+^ mice were also UM-CLL/SLL clones. Moreover, we found several *Traf2*DNx*BCL2*-tg^+/+^ UM-CLL/SLL clones expressing HCDR3 highly similar to HCDR3 recognizing autoantigens (phosphatidylcholine) and pathogen antigens (CMV, hepatitis C virus, and lipoteichoic acid). However, HCDR3 with similar antigen specificities were also found in M-CLL/SLL clones, recognizing autoantigens, such as cardiolipin and dsDNA, and pathogen antigens (*Bordetella*) (**Table 3**). In this regard, Herve and coworkers (12) have shown that both M- and UM-CLL clones derived from self-reactive B cell precursors and our data would be in agreement with those results.

Finally, the presence of B cell clones with similar HCDR3 sequence in mice with different genotypes (*Traf2*DNx*BCL2*-tg^-/-^; ^+/-^, ^-/+^ and ^+/+^) suggests that all mice are exposed to similar antigens and have similar immune responses to them. Exposure to the same antigens should be expected considering that mice in this study are littermates and are housed together. The fact that only the *Traf2*DNx*BCL2*-tg^+/+^ mice develop CLL/SLL highlights the need of both *Traf2* deficiency and BCL2 overexpression for promoting CLL development in this mouse model and underlines a role for autoantigens- and pathogen antigens-specific HCDR3 in driving disease progression.

## Data Availability Statement

The raw data supporting the conclusions of this article will be made available by the authors, without undue reservation.

## Ethics Statement

The animal study was reviewed and approved by Bioethics Committee of the Consejo Superior de Investigaciones Científicas (CSIC).

## Author Contributions

GP-C designed, performed, and analyzed the experiments and helped writing the paper. JZ designed and analyzed the experiments and wrote the paper. All authors contributed to the article and approved the submitted version.

## Funding

This work was supported by grants from the Agencia Estatal de Investigacion (PID2019-110405RB-I00/AEI/10.13039/501100011033) and from the Instituto de Salud Carlos III (PI16/000895). We acknowledge support of the publication fee by the CSIC Open Access Publication Support Initiative through its Unit of Information Resources for Research (URICI). The cost of this publication was paid in part by FEDER funds.

## Conflict of Interest

The authors declare that the research was conducted in the absence of any commercial or financial relationships that could be construed as a potential conflict of interest.
