# Supplementary material for: The Traf2DNxBCL2-tg Mouse Model of Chronic Lymphocytic Leukemia/Small Lymphocytic Lymphoma Recapitulates the Biased IGHV Gene Usage, Stereotypy, and Antigen-Specific HCDR3 Selection of Its Human Counterpart
Source: Front Immunol. 2021 Apr 12;12:627602. doi: 10.3389/fimmu.2021.627602 (PMC8072112; doi:10.3389/fimmu.2021.627602)
Supplement: Supplementary file 1 [file DataSheet_1.pdf]

SUPPLEMENTARY INFORMATION

The *Traf2*DNx*BCL2-tg* mouse model of Chronic Lymphocytic Leukemia/Small Lymphocytic Lymphoma recapitulates the biased IGHV gene usage, stereotypy and antigen-specific HCDR3 selection of its human counterpart.

Gema Perez-Chacon and Juan M. Zapata

Supplementary Materials and Methods

Criteria for assigning sequence variations differing from the GL sequence as strain-specific polymorphism (SSP) or somatic hypermutation (SHM)

The GL sequences stored in the IMGT repertoire IG database are mostly based on the C57BL/6 strain, although a few sequences from the 129/sv and BALB/c lines are also included (30). However, the *Traf2*DNx*BCL2*-tg mice are FVB/N x BALB/c F1 hybrids. Since BALB/c sequences are underrepresented and FVB/N IGHV sequences are absent in the IMGT database, this raises the question on whether nucleotide mismatches with the IMGT referenced GL sequence are the result of SSP or SHM. An accurate identification of SHM events in the IGHV sequence of a B cell clone would require the comparison with the corresponding GL sequence of the mouse strain but this is not available for the FVB/N strain. However, when comparing several IGHV sequences from B cell clones expressing the same IGHV gene, SSP would be characterized by the majority presence of one particular nucleotide different to that in the GL reference, while SHM is a random process that could introduce any nucleotide in a given position. Thus, to discriminate between SSP and SHM, we have compared the available sequences (n≥3) for a given IGHV gene from FVB/N x BALB/c F1 hybrid B cell clones (isolated from *Traf2*DNx*BCL2*-tg and *Traf3*x*BCL2*-tg mice irrespective of their genotype) with the corresponding IMGT GL sequence using a clustal W multiple sequence analysis tool. We found distinct cases and the criteria applied to discriminate between SSP, SHM or to label the mismatch as uncertain were the follow:

Case 1) When the number of genes compared (n) was ≥3 and ≤4, SSP was assigned if only one nucleotide differing from the GL sequence was found in 75% of the analyzed sequences.

Case 2) When the number of genes compared (n) was ≥5, SSP was assigned if the same mismatching nucleotide found in 70% of the analyzed genes, that is, 4 out of 5, 5 out of 7, 6 out of 8 and so on).

Case 3) When the number of genes compared (n) was ≥5, SSP was assigned if the same mismatching nucleotide was found in ≥40% of the analyzed genes as long as only 2 nucleotides were found in that position.

Case 4) When the number of genes compared (n) was ≥5, if the same mismatching nucleotide was found in ≥40% but <70% of the genes but ≥3 different nucleotides were found in that position, we did not decide whether the most frequent mismatch was SSP or SHM and labeled it as uncertain.

All other mismatches with the GL sequence were considered SHM.

SUPPLEMENTARY TABLE 1. Characteristics of representative B cell clones from the *Traf2*DNx*BCL2*-tg$^{-/-}$ (wild-type) mice.

In the table is indicated the mouse ID number, the tissue source of the mRNA sample, the age and the sex of the mice. The immunoglobulin IGHV, IGHD and IGHJ subgroups and genes found recombined in each B cell clone are indicated, according to IMGT/V-QUEST and Vbase2 analysis tools. The percentage of sequence identity of the IGHV gene respective to the IMGT referenced GL sequence is indicated (% identity), as well as the % of variation likely corresponding to strain-specific polymorphisms (SSP %) and somatic hypermutation (SHM %). The Uncertain column indicates the % of sequence variation with the referenced GL IGHV gene that could not be assigned to SSP or SHM (Supplementary Materials and Methods). SHM status indicates whether the IGHV region is unmutated (UM; ≤ 2% difference from the GL sequence) or mutated (M; >2% difference from the GL sequence). * indicates that no assessment of SSP and SHM could be done due to the low representation of those genes (n<3) in the whole *Traf2*DNx*BCL2*-tg B cell clone cohort. The frequency and % of occurrence of the B cell clones isolated from the spleens of each mouse is also shown. All clones encoded a productive Ig and the HCDR3 sequence is also provided. Basic (red) and acid (green) amino acids are highlighted and the amino acid length and isoelectric point (pI) of the HCDR3 sequence are shown.

Animal no.	sex	age months	Tissue	IGHV subgroup IMGT	IGHV gene IMGT	IGHV gene Vbase2	IGD subgroup IMGT	IGD gene	IGHJ gene	GL IMGT identity (%)	SSP %	uncertain %	SHM %	SHM status	Frequency	%	HCDR3	HCDR3 lenght	pI
TRAF2DNxBCL2 8 -/-	M	14	Spleen	VH6	IGHV6-6*02 F	VHJ606.a6.127	D4	DQ52	JH2	97.9	1.7	-	0.35	UM	2/10	20	TSWDVNY	7	3.8
				VH2	IGHV2-4-1*01 F	VHQ52.a12.33	D2	DSP2.x	JH4	94.0	1.4	-	4.6	M	1/10	10	ARKGRTYYSNHGAMDY	16	9.53
				VH14	IGHV14-1*02 F	VHSM7.a1.83	D2	DSP2.12	JH3	96.9	1.1	-	2	UM	1/10	10	ARSYDGFAY	9	5.88
				VH14	IGHV14-3*02 F	VHSM7.a3.93	D6	PseudoD2	JH2	95.1	3.1	-	1.7	UM	1/10	10	GRHSVYYFDY	10	6.74
				VH14	IGHV14-3*02 F	VHSM7.a3.93	D1	DFL16.1	JH2	92.0	1.4	.-	6.6	M	1/10	10	TRDYGSGYFAF	11	5.5
				VH2	IGHV2-9-1*01	VHQ52.a27.79	D2	DSP2.13	JH2	86.7	1	-	12.3	M	1/10	10	ARFGDYGGWDLDY	13	3.93
				VH14	IGHV14-3*02 F	VHSM7.a3.93	D2	DSP2.5	JH3	98.3	1.0	.-	0.7	UM	1/10	10	ASLRFAY	7	8.79
				VH3	IGHV3-8*02 F	VH36-60a8.117	D2	DSP2.2	JH4	90.2	0.7	-	9.1	M	1/10	10	AKWMGAYYDYDVGYGVDH	18	4.41
				VH1	IGHV1-80*01 F	VHJ558.83.189	D1	DFL16.1	JH2	87.8	3.5	-	8.7	M	1/10	10	ARSPGFKYGDY	11	8.54
TRAF2DNxBCL2 14 -/-	M	13	Spleen	VH11	IGHV11-2*02	VH11.a2.92	D2	DSP2.5	JH1	98.3	1.7	-	0	UM	1/15	6.6	MRYGNYWYFDV	11	5.59
				VH11	IGHV11-2*02	VH11.a2.92	D2	DSP2.4	JH1	98.3	1.4	-	0.35	UM	1/15	6.6	MRYGYGYWYFDV	12	5.59
				VH5	IGHV5-17*02	VH7183.a47.76	D2	DSP2.6	JH4	100	0	-	0	UM	1/15	6.6	ATYYGYDRVYYYAMDY	16	4.21
				VH11	IGHV11-2*02	VH11.a2.92	D3	DST4.3	JH1	98.3	1.7	-	0	UM	1/15	6.6	IRYDRYWYFDV	11	5.96
				VH11	IGHV11-2*02	VH11.a2.92	D2	DSP2.2	JH1	98.3	1.7	-	0	UM	1/15	6.6	MRYSSYDYDWYFDV	14	3.93
				VH3	IGHV3-2*02	VH36-60-a2.90	D2	DSP2.5	JH4	95.8	unknown	-	4.2	M*	1/15	6.6	ARHYGNYYAMDY	12	6.78
				VH1	IGHV1S130*01	VHJ558.f	D1	DFL16.1e	JH2	97.6	2.1	-	0.35	UM	2/15	13.2	ASGYFDY	7	3.8
				VH4	IGHV4-1*02	VHX24.a1.84	D1	DFL16.1	JH2	97.6	2.1	-	0.35	UM	1/15	6.6	ARLSYYGRSDF	11	8.63
				VH2	IGHV2-9*02	VHQ52.a13.37	D4	DQ52	JH4	99.0	0	0.35	0.7	UM	1/15	6.6	ASYWDARDY	9	4.21
				VH1	IGHV1-9*01	VHJ558.b9	D2	DSP2.2	JH2	88.5	1.74	-	9.7	M	1/15	6.6	ARYDYDEGFYFDY	13	3.84
				VH2	IGHV2-4-1*01	VHQ52.b4	D2	DSP2.5	JH2	96.8	1.4	-	1.8	UM	1/15	6.6	AREDGNYVHFGY	12	5.32
				VH2	IGHV2-6-7*01	VHQ52.a24.72	D2	DSP2.2	JH4	96.4	1.1	-	2.5	M	1/15	6.6	ARIYYDYDGNYVMDY	15	3.93
				VH1	IGHV1S34*01	IGHV643	Unknown	Unknown	JH4	91.5	unknown	-	8.5	M*	1/15	6.6	AREDFGHDVRTMDY	14	4.66
				VH1	IGHV1-4*01	VHJ558.b4	D2	DSP2.7	JH2	93.4	unknown	-	6.6	M*	1/15	6.6	ARAAHYEPYFFDY	13	5.32
TRAF2DNxBCL2 35 -/-	M	20	Spleen	VH2	IGHV2-5*01 F	VHQ52.a7.18	D1	DFL16.1	JH4	97.6	unknown	-	2.5	M*	1/11	9	AKEDYYGSSYCYAMDY	16	4.03
				VH1	IGHV1-14*01 P	VHJ558.b14	D2	DSP2.7	JH3	90.7	2.4	-	6.9	M	1/11	9	ATYYGNAWFAY	11	5.57
				VH1	IGHV1S82*01 [F]	VH468	Unknown	Unknown	JH2	97.2	unknown	-	2.8	M*	1/11	9	CARYYYGSNFDY	12	5.83
				VH3	IGHV3-8*02 F	VH36-60.a8.117	D1	DFL16.1	JH1	97.5	0.7	-	1.8	UM	1/11	9	YYGSSYWYFDV	11	3.8
				VH2	IGHV2-5*01	VHQ52.a7.18	Unknown	Unknown	JH1	98.1	unknown	.-	1.9	UM	1/11	9	ARGDMITTRYFDV	13	6
				VH5	IGHV5-12-1*01 F	VH7183.a28.48	D3	DST4.3	JH4	95.8	1	-	3.1	M	2/11	18	ARQGAMDY	8	5.88
				VH6	IGHV6-6*01 F	VHJ606.b5	D1	DFL16.1	JH2	94.6	1.0	-	4.4	M	2/11	18	TSHDYGSTYRGYYFDY	16	5.18
				VH1	IGHV1-39*01 F	VHJ558.b42	D2	DSP2.9	JH4	91.3	2.7	0.35	5.5	M	1/11	9	ATGDYYTVEY	10	3.67
				VH1	IGHV1-69*02 F	VH124	D2	DSP2.9	JH3	92.7	2.4	0.35	4.5	M	1/11	9	TADDYLFAY	9	3.56

**TRAF2DNxBCL2 37 -/-**	**37 -/- #1**	**M**	**20**	**Spleen**	VH1	IGHV1S135*01 [F]	VHJ558.1	D2	DSP2.5	JH1	98.6	0.35	-	1.0	UM	1/10	10	ARSGNYWYFDV	11	5.88
	**37 -/- #2**				VH6	IGHV6-6*02 F	VHJ606.a6.127	Unknown	Unknown	JH2	99	1.0	-	0	UM	1/10	10	CTGSDY	6	3.8
	**37 -/- #3**				VH2	IGHV2-9*02 F	VHQ52.a13.37	D2	DQ52	JH4	96.1	0.7	-	3.2	M	1/10	10	ARPTMITDYSMDY	13	4.21
	**37 -/- #4**				VH4	IGHV4-1*02 F	VHX24.a1.84	D3	DST4.3	JH2	95.5	3.9	-	0.7	UM	1/10	10	ATSWDSGY	8	3.8
	**37 -/- #5**				VH3	IGHV3-1*02 F	VH36-60.a1.85	D2	DSP2.13	JH4	95.2	3.8	-	1.1	UM	1/10	10	ARRWGRDAMDY	11	8.79
	**37 -/- #6**				VH14	IGHV14-1*02 F	VHSM7.a1.83	D2	DSP2.9	JH2	93.4	1.1	-	5.5	M	2/10	20	ARFTTMVTTWLFDY	14	5.88
	**37 -/- #7**				VH1	IGHV1-18*01 F	VHJ558.b19	D2	DSP2.12	JH4	96.2	unknown	-	3.8	M*	1/10	10	ARRYFGSFLYYAMDY	15	8.47
	**37 -/- #8**				VH3	IGHV3-1*02 F	VH36-60.a1.85	D2	DSP2.2	JH4	94.1	4.2	.-	1.7	UM	1/10	10	ARDYDYDGGN	10	3.93
	**37 -/- #9**				VH7	IGHV7-3*02 F	VHS107.a3.106	D1	DFL16.1	JH1	94.2	1	-	4.8	M	1/10	10	ARDIVIYGYGYFDV	14	4.21
**TRAF2DNxBCL2 47 -/-**	**47 -/- #1**	**F**	**19**	**Spleen**	VH11	IGHV11-2*02	VH11.a2.92	D2	DSP2.2	JH1	98.0	1.7	-	0.35	UM	1/11	9	MRYSSYDYDWYFDV	14	3.93
	**47 -/- #2**				VH5	IGHV5-6-3*01	VH7183.a30.50	D1	DFL16.1	JH3	95.8	0	-	4.2	M	1/11	9	ARDYHYGSSRFAY	13	8.55
	**47 -/- #3**				VH2	IGHV2-9-1*01	VHQ52.a27.79	D2	DSP2.5	JH3	98.6	1		0.35	UM	1/11	9	ASLLLPFAY	9	5.57
	**47 -/- #4**				VH1	IGHV1-62-2*01 F	VHJ558.b67	D1	DFL16.1	JH3	92.4	0.35	-	7.3	M	1/11	9	ARHEDPYFYEGSCFAY	16	4.65
	**47 -/- #5**				VH1	IGHV1-87*01	VH021	D2	DSP2.5	JH4	96.5	2.1	-	1.4	UM	1/11	9	ARMNGNYVAMDY	12	5.88
	**47 -/- #6**				VH1	IGHV1S56*01	VH43Y	Unknown	Unknown	JH2	96.9	1.4	-	1.7	UM	1/11	9	ARSLTGTEDY	10	4.37
	**47 -/- #7**				VH5	IGHV5-17*02	VH7183.a47.76	D1	DFL16.2	JH3	94.8	1.7	0.35	3.1	M	1/11	9	ARSGGLITTAMVFTY	15	8.79
	**47 -/- #8**				VH14	IGHV14-3*02	VHSM7.a3.93	D2	DSP2.4	JH2	96.5	1.4	-	2.1	M	1/11	9	ARRVGLPYYFDY	12	8.54
	**47 -/- #9**				VH14	IGHV14-3*02	VHSM7.a3.93	D2	DSP2.4	JH2	95.8	1.4	-	2.7	M	1/11	9	ARSSGYYYGYDGYYFDY	17	4.21
	**47 -/- #10**				VH1	IGHV1S33*01	VH049	Unknown	Unknown	JH1	95.2	unknown	-	4.8	M*	1/11	9	AREGGGNWYFDV	12	4.37
	**47 -/- #11**				VH1	IGHV1S135*01	VHJ558.1	D1	DFL16.3	JH4	93.8	0.35	-	6.3	M	1/11	9	GRAPRSSPYAMDY	13	8.59
**TRAF2DNxBCL2 60 -/-**	**60 -/- #1**	**F**	**18**	**Spleen**	VH11	IGHV11-2*02	VH11.a2.92	D1	DFL16.1	JH2	98.3	1.7	-	0	UM	1/12	8.3	MRYGSYWYFDV	11	5.59
	**60 -/- #2**				VH11	IGHV11-2*02	VH11.a2.92	D2	DSP2.5	JH1	98.3	1.7	-	0	UM	1/12	8.3	MRYGNYWYFDV	11	5.59
	**60 -/- #3**				VH5	IGHV5-12-1*01	VH7183.a28.48	D3	DST4	JH3	97.9	1.4	-	0.7	UM	1/12	8.3	ARDSFAY	7	5.88
	**60 -/- #4**				VH5	IGHV5-12-1*01	VH7183.a28.48	D4	DQ52	JH2	98.3	1.4	-	0.35	UM	1/12	8.3	ARHGRGY	7	10.84
	**60 -/- #5**				VH1	IGHV1S81*02	VHJ558.c	Unknown	Unknown	JH4	94.5	1.7	-	3.8	M	1/12	8.3	TRSPFITTVVATSDAMDY	18	4.21
	**60 -/- #6**				VH5	IGHV5-17*02	VH7183.a47.76	D4	DQ52	JH2	97.9	1.4	0.7	0	UM	1/12	8.3	ARSGNWDVVRGDY	13	6
	**60 -/- #7**				VH1	IGHV1-39*01	VHJ558.b42	D1	DFL16.1e	JH2	88.2	2.4	0.35	9	M	1/12	8.3	ARSGGNYNFDY	11	5.88
	**60 -/- #8**				VH5	IGHV5-9*02	VH7183.a16.27	D2	DSP2.5	JH2	93.0	2.1	-	4.9	M	1/12	8.3	ARHNYGNSYYFES	13	6.79
	**60 -/- #9**				VH1	IGHV1S135*01	VHJ558.1	D1	DFL16.1	JH4	90.2	0.35	-	9.4	M	1/12	8.3	ASHYYGSGGYAMDY	14	5.08
	**60 -/- #10**				VH5	IGHV5-9*02	VH7183.a10.15	D2	DSP2.5	JH2	92.0	2.5	-	5.6	M	1/12	8.3	ARHNYGNSYYFES	13	6.79
	**60 -/- #11**				VH1	IGHV1-67*01	VHJ558.b73	D2	DSP2.2	JH3	93.1	4.2	-	2.8	M	1/12	8.3	ARGRDYDAWFTY	12	6
	**60 -/- #12**				VH1	IGHV1-15*01	VHJ558.b15	D1	DFL16.2	JH3	94.8	0.7	-	4.5	M	1/12	8.3	TRDDSPLRFTY	11	5.63
**TRAF2DNxBCL2 71 -/-**	**71 -/- #1**	**F**	**15**	**Spleen**	VH1	IGHV1S130*01 [F]	VHJ558.f	D1	DFL16.1e	JH2	95.8	4.2	-	0	UM	1/7	14	ARGYGDYFDY	10	4.21
	**71 -/- #2**				VH1	IGHV1-69*02 F	VH124	D1	DFL16.1	JH3	91.7	3.5	0.35	4.5	M	2/7	28	ARVYYDGSPWFAY	13	5.88
	**71 -/- #3**				VH3	IGHV3-6*01 F	VH36-60.b6	D2	DSP2.8	JH4	93.4	4.2	-	2.4	M	1/7	14	AGDILGYYAMDY	12	3.56
	**71 -/- #4**				VH1	IGHV1-74*04 F	VH102	D2	DSP2.9	JH3	91.7	6.9	-	1.4	UM	1/7	14	YTMPATGAWFAY	12	5.52
	**71 -/- #5**				VH1	IGHV1-81*01 F	VHJ558.84.190	D2	DSP2.x	JH2	95.8	2.4	-	1.7	UM	1/7	14	ARDSNYVGFDY	11	4.21
	**71 -/- #6**				VH1	IGHV1-69*02 F	VH124	D1	DFL16.1	JH3	92.4	2.8	0.35	4.5	M	1/7	14	ARVYYDGSPWFAC	13	5.87

SUPPLEMENTARY TABLE 2. Characteristics of representative B cell clones from the *Traf2*DNx*BCL2*-tg$^{+/-}$ (*Traf2*DN-tg) mice.
In the table is indicated the mouse ID number, the tissue source of the mRNA sample, the age and the sex of the mice. The immunoglobulin IGHV, IGHD and IGHJ subgroups and genes found recombined in each B cell clone are indicated, according to IMGT/V-QUEST and Vbase2 analysis tools. The percentage of sequence identity of the IGHV gene respective to the IMGT referenced GL sequence is indicated (% identity), as well as the % of variation likely corresponding to strain-specific polymorphisms (SSP %) and somatic hypermutation (SHM %). The Uncertain column indicates the % of sequence variation with the referenced GL IGHV gene that could not be assigned to SSP or SHM (Supplementary Materials and Methods). SHM status indicates whether the IGHV region is unmutated (UM; ≤ 2% difference from the GL sequence) or mutated (M; >2% difference from the GL sequence). * indicates that no assessment of SSP and SHM could be done due to the low representation of those genes (n<3) in the whole *Traf2*DNx*BCL2*-tg B cell clone cohort. The frequency and % of occurrence of the B cell clones isolated from the spleens of each mouse is also shown. All clones encoded a productive Ig and the HCDR3 sequence is also provided. Basic (red) and acid (green) amino acids are highlighted and the amino acid length and isoelectric point (pI) of the HCDR3 sequence are shown.

Animal no.		sex	months	Tissue	IGHV subgroup IMGT	IGHV gene IMGT	IGHV gene Vbase2	IGD subgroup IMGT	IGD gene	IGHJ gene	GL IMGT identity (%)	SSP %	uncertain %	SHM %	SHM status	Frequency	%	HCDR3	HCDR3 lenght	pI
TRAF2DNxBCL2 11+/-	11+/- # 1	M	13	Spleen	VH1	IGHV1-5*01 F	VHJJ58.b5	D2	DSP2.11	JH4	98.1	0	-	1.9	UM	1/10	10	TRYRYEDYAMDY	12	4.56
	11+/- # 2				VH1	IGHV1S81*02 [F]	VHJ558.c	Unknown	Unknown	JH4	97.2	2.1	-	0.7	UM	1/10	10	ARQNAMDY	8	5.88
	11+/- # 3				VH1	IGHV1S81*02 [F]	VHJ558.c	Unknown	Unknown	JH4	98.3	1.7	-	0	UM	1/10	10	ARSNAMDY	8	5.88
	11+/- # 4				VH14	IGHV14-3*02 F	VHSM7.a3.93	D1	DFL16.3	JH4	97.9	1.4	-	0.7	UM	1/10	10	ARGGKGGYYAMDY	13	8.47
	11+/- # 5				VH2	IGHV2-2*02 F	VHQ52.a2.4	D2	DFL16.3	JH4	98.6	0	-	1.4	UM	1/10	10	ARRWYDGYYAMDY	13	6
	11+/- # 6				VH2	IGHV2-2*02 F	VHQ52.a2.4	Unknown	Unknown	JH2	97.9	0	-	2.1	M	1/10	10	ARTDY	5	5.88
	11+/- # 7				VH5	IGHV5-9-1*01 F	VH7183.a16.24	D1	DFL16.1	JH4	98.6	unknown	-	1.4	UM	2/10	20	ASSYYYAMDY	10	3.8
	11+/- # 8				VH1	IGHV1S81*02 [F]	VHJ558.c	Unknown	Unknown	JH2	97.9	1.7	.	0.35	UM	2/10	20	AHLGRGYFDY	10	6.79
TRAF2DNxBCL2 24+/-	24+/- # 1	M	20	Spleen	VH6	IGHV6-6*02 F	VHJ606.a6.127	D4	DQ52	JH2	98.0	1.7	-	0.35	UM	1/6	16.6	TSWDVNY	7	3.8
	24+/- # 2				VH1	IGHV1S130*01 [F]	VHJ558.f	D2	DSP2.12	JH3	95.1	4.2	-	0.7	UM	1/6	16.6	ARGVYSYSTFAY	12	8.54
	24+/- # 3				VH1	IGHV1S130*01 [F]	VHJ558.f	D6	P7	JH3	96.9	2.1	-	1	UM	1/6	16.6	AREDDGRRGFAY	12	6.17
	24+/- # 4				VH2	IGHV2-9*02 F	VHQ52.a13.37	D2	DSP2.11	JH3	97.9	1.1	0.35	0.7	UM	1/6	16.6	ASSYYRYPFAY	11	8.47
	24+/- # 5				VH5	IGHV5-12-2*01 F	VH7183.a35.57	D2	DSP2.13	JH4	98.3	1.4	-	0.35	UM	1/6	16.6	ARRRGDRAMDY	11	10.67
	24+/- # 6				VH1	IGHV1-69*01 F	VHJ558.72.173	D1	DFL16	JH2	94.4	2.8	-	2.8	M	1/6	16.6	ARRYYDGSYGYFDY	14	6
TRAF2DNxBCL2 58+/-	58+/- # 1	F	18	Spleen	VH1	IGHV1-80*01 F	VHJ558.83.189	D2	DSP2.9	JH2	94.8	3.8	-	1.4	UM	1/8	13	AREGDGYYDFDY	12	3.84
	58+/- # 2				VH1	IGHV1-9*01 F	VHJ558.b9	D2	DSP2.2	JH3	90.6	1.4	0.35	7.6	M	1/8	13	ARKDYDYLFAY	11	6
	58+/- # 3				VH1	IGHV1S130*01 [F]	VHJ558.f	D3	DST4	JH3	95.5	3.5	-	1	UM	1/8	13	ARGNPEGFAY	10	6.05
	58+/- # 4				VH6	IGHV6-6*02 F	VHJ606.a6.127	D6	P7	JH4	97.6	1.4	-	1	UM	1/8	13	TREALYYYAMDY	12	4.37
	58+/- # 5				VH1	IGHV1S81*02 [F]	VHJ558.c	Unknown	Unknown	JH2	93.7	2.1	-	4.2	M	1/8	13	TRRSYYSYDAGDY	13	5.63
	58+/- # 6				VH6	IGHV6-6*02 F	VHJ606.a6.127	D4	DQ52	JH2	98.3	1	-	0.7	UM	1/8	13	TSWDVNY	7	3.8
	58+/- # 7				VH1	IGHV1-74*04 F	VH102	D3	DST4.2	JH3	88.9	9.7	-	1.4	UM	1/8	13	ARKEAPSGSFAY	12	8.63
	58+/- # 8				VH12	IGHV12-3*01 F	VH12.b3	D4	DQ52	JH1	97.3	1	-	1.7	UM	1/8	13	AGDSTGYWYFDV	12	3.56
TRAF2DNxBCL2 68+/-	68+/- # 1	F	15	Spleen	VH2	IGHV2-9*02 F	VHQ52.a13.37	D2	DSP2.11	JH3	97.8	1.1	0.35	0.7	UM	1/5	20	ASSYYRYPFAY	11	8.47
	68+/- # 2				VH3	IGHV3-6*02 F	VH36-60.a6.114	D2	DSP2.2	JH4	98.3	1.4	-	0.35	UM	1/5	20	ARDPTPIYYDYDVEAMDY	18	3.71
	68+/- # 3				VH1	IGHV1-69*02 F	VH124	D2	DSP2.12	JH3	91.3	2.8	-	5.9	M	1/5	20	TREDDGFAY	9	4.03
	68+/- # 4				VH5	IGHV5-12-2*01 F	VH7183.a35.57	D4	DQ52	JH4	98.6	1.4	-	0	UM	1/5	20	ARTLGAMDY	9	5.88
	68+/- # 5				VH1	IGHV1-9*01 F	VHJ558.b9	D4	DQ52	JH3	96.9	2.4	0.35	0.35	UM	1/5	20	ARGGLGAY	8	8.79
	68+/- # 6				VH5	IGHV5-17*02 F	VH7183.a47.76	D2	DST2.5	JH4	97.6	1.4	0.7	0.35	UM	1/5	20	ASNYVGAMDY	10	3.8

SUPPLEMENTARY TABLE 3. Characteristics of representative B cell clones from the *Traf2*DNx*BCL2*-tg$^{-/+}$ (*BCL2*-tg) mice.
In the table is indicated the mouse ID number, the tissue source of the mRNA sample, the age and the sex of the mice. The immunoglobulin IGHV, IGHD and IGHJ subgroups and genes found recombined in each B cell clone are indicated, according to IMGT/V-QUEST and Vbase2 analysis tools. The percentage of sequence identity of the IGHV gene respective to the IMGT referenced GL sequence is indicated (% identity), as well as the % of variation likely corresponding to strain-specific polymorphisms (SSP %) and somatic hypermutation (SHM %). The Uncertain column indicates the % of sequence variation with the referenced GL IGHV gene that could not be assigned to SSP or SHM (Supplementary Materials and Methods). SHM status indicates whether the IGHV region is unmutated (UM; ≤ 2% difference from the GL sequence) or mutated (M; >2% difference from the GL sequence). * indicates that no assessment of SSP and SHM could be done due to the low representation of those genes (n<3) in the whole *Traf2*DNx*BCL2*-tg B cell clone cohort. The frequency and % of occurrence of the B cell clones isolated from the spleens of each mouse is also shown. All clones encoded a productive Ig and the HCDR3 sequence is also provided. Basic (red) and acid (green) amino acids are highlighted and the amino acid length and isoelectric point (pI) of the HCDR3 sequence are shown.

Animal no.	sex	months	Tissue	IGHV subgroup IMGT	IGHV gene IMGT	IGHV gene Vbase2	IGD subgroup	IGD gene	IGHJ gene	GL IMGT identity (%)	SSP %	uncertain %	SHM %	SHM status	Frequency	%	HCDR3	HCDR3 lenght	pI
TRAF2DNxBCL2 17 -/+	M	13	Spleen	VH1	IGHV1-9*01	VHJ558.b9	D2	DSP2.4	JH4	91.7	1.74	-	6.6	M	1/15	6.7	ARGGYYGYDGDYYAMDY	17	3.93
				VH5	IGHV5-17*02	VH7183.a47.76	D1	DFL16.1	JH2	100	0	-	0	UM	1/15	6.7	ARSSSSYFDY	10	5.88
				VH1	IGHV1-12*01	VHJ558.b12	D1	DFL16.1	JH1	82.2	unknown	-	17.8	M*	1/15	6.7	TRSDYGRMHDWCFDV	15	5.27
				VH1	IGHV1S56*01 F	VH43Y	Unknown	Unknown	JH2	96.7	1.4	-	1.7	UM	1/15	6.7	ARGRYGDY	8	8.63
				VH2	IGHV2-6-2*01 F	VHQ52.a15.42	D4	DQ52	JH4	96.8	2.1	-	1.1	UM	1/15	6.7	ARELGRRYAMDY	12	8.63
				VH1	IGHV1-81*01 F	VHJ558.84.190	D2	DSP2.x	JH2	96.9	2.1	-	1.1	UM	1/15	6.7	ARGAVYSNQYYFDY	14	5.88
				VH14	IGHV14-3*02 F	VHSM7.a3.93	D1	DFL16.3	JH2	95.1	1	-	3.8	M	1/15	6.7	AVERGAGYYFDY	12	4.37
				VH6	IGHV6-6*02 F	VHJ606.a6.127	D4	DQ52	JH3	98.3	1.4	-	0.35	UM	1/15	6.7	TTGTFAY	7	5.18
				VH2	IGHV2-2*01 F	VHQ52.b1	D2	DSP2.x	JH2	97.2	0.7	0.35	1.7	UM	1/15	6.7	ARSYYSKYERLYFDY	15	8.38
				VH5	IGHV5-17*02 F	VH7183.a47.76	D3	DST4.2	JH2	98.3	0.7	0.7	0.35	UM	1/15	6.7	ARSGKGFDY	9	8.63
				VH1	IGHV1-80*01 F	VHJ558.83.189	D4	DQ52	JH3	93.8	3.5	-	2.7	M	1/15	6.7	ARRELGRAWFAY	12	10.74
				VH2	IGHV2-2*01 F	VHQ52.b1	D3	DST4.3	JH2	96.9	1	0.35	1.7	UM	1/15	6.7	ARNGGQVWYYFDY	13	5.88
				VH2	IGHV2-9*02 F	VHQ52a13.37	D2	DSP2.9	JH3	96.5	1.1	0.35	2.1	M	1/15	6.7	ARDDAYY	7	4.21
				VH1	IGHV1-62-2*01 F	VHJ558.b67	D2	DSP2.9	JH3	97.6	0.35	-	2.1	M	1/15	6.7	ARHEESEGNYWFAY	14	4.75
				VH1	IGHV1S136*01 [F]	VHJ558.m	Unknown	Unknown	JH2	93.8	unknown	-	6.2	M*	1/15	6.7	AREGIHDNYYFDF	13	4.54
TRAF2DNxBCL2 64 -/+	F	18	Spleen	VH2	IGHV2-6-4*01 F	VHQ52.a19.61	D2	DSP2.5	JH3	98.1	unknown		1.9	UM	2/10	20	ASYYGNYWFAY	11	5.57
				VH5	IGHV5-17*02	VH7183.a47.76	D2	DSP2.6	JH4	100	0	-	0	UM	2/10	20	ATYYGYDRVYYYAMDY	16	4.21
				VH2	IGHV2-6-7*01 F	VHQ52.a24.72	D1	DFL16.1e	JH3	98.6	1.1	-	0.35	UM	1/10	10	ARDGFAY	7	5.88
				VH2	IGHV2-6-7*01 F	VHQ52.a24.72	D1	DFL16.1	JH4	98.2	1.1	-	0.7	UM	1/10	10	ATRPYYYGREGYYGMDY	17	6.11
				VH1	IGHV1-14*01 P	VHJ558.b14	D6	P7	JH1	96.9	2.7	-	0.35	UM	1/10	10	ARCGDFSYWYFDV	13	4.21
				VH7	IGHV7-3*02 F	VHS107.a3.106	D2	DSP2.11	JH4	93.9	1.7		4.4	M	1/10	10	ARDAYYRYAELPFWYAMDY	19	4.56
				VH7	IGHV7-3*02 F	VHS107.a3.106	D2	DSP2.8	JH3	97.6	1.7	-	0.7	UM	1/10	10	ARDMGYGAWFAY	12	5.88
				VH2	IGHV2-6-7*01 F	VHQ52.a24.72	D4	DQ52	JH2	97.5	0		2.5	M	1/10	10	ARDRWGYFDY	10	6
TRAF2DNxBCL2 79 -/+	F	15	Spleen	VH5	IGHV5-17*02	VH7183.a47.76	D2	DSP2.5	JH4	98.3	0	-	1.74	UM	1/12	8.3	ARELYDGSYYYAMDY	15	4.03
				VH5	IGHV5-6-3*01	VH7183.a30.50	D2	DSP2.6	JH4	99.0	0	-	1	UM	1/12	8.3	ATYYGYDRVYYYAMDY	16	4.21
				VH1	IGHV1S135*01 [F]	VHJ558.1	D2	DSP2.9	JH1	94.1	0	0.35	5.9	M	1/12	8.3	ARRDDGYWYFDV	12	4.43
				VH1	IGHV1-9*01 F	VHJ558.b9	D1	DFL16	JH3	96.5	2.4	-	1	UM	1/12	8.3	ARNRPHYYDGSYLFAY	16	8.48
				VH7	IGHV7-3*02 F	VHS107.a3.106	Unknown	Unknown	JH2	98.3	1	-	0.7	UM	1/12	8.3	ARANFDY	7	5.88
				VH6	IGHV6-6*02 F	VHJ606.a6.127	Unknown	Unknown	JH3	98.3	1.4	-	0.35	UM	1/12	8.3	TTGFAY	6	5.18
				VH1	IGHV1S55*01 F	VH697	D1	DFL16	JH2	95.1	unknown	-	4.9	M*	1/12	8.3	ARYYDGSYFDY	11	4.21
				VH5	IGHV5-12-2*01 F	VH7183.a35.57	D1	DFL16.2	JH4	96.9	1.7	-	1.4	UM	1/12	8.3	ARHDYGYSYYAMDY	14	5.21
				VH1	IGHV1-67*01 F	VHJ558.b73	D2	DSP2.9	JH4	92.7	3.8	-	3.5	M	1/12	8.3	ARDGFYAMDY	10	4.21
				VH1	IGHV1-74*04 F	VH102	D1	DFL16.1e	JH1	91.7	6.6	-	3.1	M	1/12	8.3	SRGGGRYFWYFDV	13	8.31
				VH1	IGHV1-67*01 F	VHJ558.b73	D2	DSP2.9	JH4	91.6	4.2	-	4.2	M	1/12	8.3	ARDGFYGMDY	10	4.21
				VH5	IGHV5-17*02 F	VH7183.a47.76	D3	DST4.3	JH2	95.5	1.74	0.35	2.4	M	1/12	8.3	ARSRAVYYFDY	11	8.54

SUPPLEMENTARY TABLE 4. Characteristics of expanded and not expanded B cell clones from the *Traf2*DNx*BCL2*-tg$^{+/+}$ mice.

In the table is indicated the mouse ID number, the tissue source of the mRNA sample, the age and the sex of the mice. The immunoglobulin IGHV, IGHD and IGHJ subgroups and genes found recombined in each B cell clone are indicated, according to IMGT/V-QUEST and Vbase2 analysis tools. The percentage of sequence identity of the IGHV gene respective to the IMGT referenced GL sequence is indicated (% identity), as well as the % of variation likely corresponding to strain-specific polymorphisms (SSP %) and somatic hypermutation (SHM %). The Uncertain column indicates the % of sequence variation with the referenced GL IGHV gene that could not be assigned to SSP or SHM (Supplementary Materials and Methods). SHM status indicates whether the IGHV region is unmutated (UM; ≤ 2% difference from the GL sequence) or mutated (M; >2% difference from the GL sequence). * indicates that no assessment of SSP and SHM could be done due to the low representation of those genes (n<3) in the whole *Traf2*DNx*BCL2*-tg B cell clone cohort. The frequency and % of occurrence of the B cell clones isolated from the spleens of each mouse is also shown. All clones encoded a productive Ig and the HCDR3 sequence is also provided. Basic (red) and acid (green) amino acids are highlighted and the amino acid length and isoelectric point (pI) of the HCDR3 sequence are shown.

Animal no.	sex	age months	Tissue	IGHV subgroup IMGT	IGHV gene IMGT	IGHV gene Vbase2	IGD subgroup IMGT	IGD gene	IGHJ gene	GL IMGT identity (%)	SSP %	uncertain %	SHM %	SHM status	frequency	%	HCDR3	HCDR3 lenght	pI
TRAF2DNxBCL2 13+/+	F	13	Spleen	VH14	IGHV14-2*02 F	VHSM7.a2psi.88	D2	DSP2.9	JH4	95.8	3.1	0.35	1.1	UM	5/13	38	GRDDGYYYAMDY	12	3.93
				VH5	IGHV5-17*02 F	VH7183.a47.76	D3	DST4.3	JH4	95.8	1.4	0.7	2.1	M	3/13	23	AREGPRRDYYAMDY	14	6.16
				VH5	IGHV5-17*02 F	VH7183.a47.76	D2	DSP2.9	JH4	97.6	1.7	0.35	0.35	UM	1/13	8	ARWLLRYYAMDY	12	8.54
				VH1	IGHV1-55*01 F	VHJ5558.b58	D4	DQ52	JH4	85.0	1.4	-	13.5	M	1/13	8	ARHDWDGGMDY	11	4.41
				VH14	IGHV14-3*02 F	VHSM7.a3.93	D1	DFL16.1	JH2	98.3	1.4	-	0.35	UM	1/13	8	ARSPYYYGSRALFDY	15	8.47
				VH1	IGHV1-7*01 F	VHJ5558.b7	D2	DSP2.8	JH4	97.6	2.1	-	0.35	UM	1/13	8	ARRGQYDYHAMDY	13	6.79
				VH3	IGHV3-5*02 F	VH36-60.a5.112	D3	DST4	JH4	97.9	1.0	-	1.0	UM	1/13	8	ARIRGGAMDY	10	8.79
TRAF2DNxBCL2 16+/+	M	12	Spleen	VH1	IGHV1-85*01 F	VHJ558.88.194	D1	DFL16.1	JH3	93.4	6.3	-	0.35	UM	6/10	60	ASYAFAY	7	5.57
				VH5	IGHV5-17*02 F	VH7183.a47.76	D2	DSP2.2	JH4	98.3	1.0	0.35	0.35	UM	3/10	30	ASRSTMIIMDY	11	5.88
				VH3	IGHV3-6*02 F	VH36.60.a6.114	D4	DQ52	JH2	97.6	1,0	-	1.4	UM	1/10	10	ARSNFYFDY	9	5.88
			Blood	VH5	IGHV5-17*02 F	VH7183.a47.76	D2	DSP2.2	JH4	97.6	1.4	0.35	0.35	UM	7/10	70	ASRSTMIIMDY	11	5.88
				VH1	IGHV1-85*01 F	VHJ558.88.194	D1	DFL16.1	JH3	93.0	6.3	-	0.7	UM	3/10	30	ASYAFAY	7	5.57
TRAF2DNxBCL2 29+/+	M	20	Spleen	VH1	IGHV1-80*01 F	VHJ558.83.189	D2	DSP2.2	JH4	94.4	3.5	.-	2.1	M	8/10	80	ASPSYDYPYYYAMDY	15	3.56
				VH7	IGHV7-3*02 F	VHS107.a3.106	D4	DQ52	JH4	98.0	2.0	-	0	UM	1/10	10	ASLNWDWYFDV	11	3.56
				VH14	IGHV14-3*02 F	VHSM7.a3.93	D2	DSP2.2	JH2	99.0	1.0	-	0	UM	1/10	10	ARSDYDRFDY	10	4.43
TRAF2DNxBCL2 40+/+	F	11	Spleen	VH5	IGHV5-17*02 F	VH7183.a47.76	D2	DSP2.6	JH4	97.9	1.4	0.7	0	UM	8/10	80	ATYYGYDRVYYYAMDY	16	4.21
				VH11	IGHV11-2*02 F	VH11.a2.92	D2	DSP2.2	JH1	96.9	0.35	-	2.8	M	1/10	10	MRYSSYDYDWYFDV	14	3.93
				VH1	IGHV1-12*01 F	VHJ558.b12	D4	DQ52	JH2	93.1	unknown	-	6.9	M*	1/10	10	ARGENWDSFDY	11	4.03
			Blood	VH5	IGHV5-17*02 F	VH7183.a47.76	D2	DSP2.6	JH4	97.9	1.4	0.35	0.35	UM	9/10	90	ATYYGYDRVYYYAMDY	16	4.21
				VH1	IGHV1-63*02 F	VHJ558.15	D2	DSP2.2	JH4	98.6	unknown	-	1.4	UM	1/10	10	ARGGDDYGSRGYAMDY	16	4.43
TRAF2DNxBCL2 45+/+	F	20	Spleen	VH1	IGHV1-9*01 F	VHJ558.b9	D2	DSP2.2	JH3	96.5	2.4	0.35	0.7	UM	8/10	80	ARGDYDGEFAY	11	4.03
				VH1	IGHV1-18*01 F	VHJ558.b19	D2	DSP2.5	JH4	97.9	unknown	-	2.1	M*	1/10	10	AYGNYAMDY	9	3.8
				VH1	IGHV1S29*02 F	VH108A	Unknown	Unknown	JH4	97.6	unknown	-	2.4	M*	1/10	10	ARYAYYGSSYNYAMDY	16	5.88
TRAF2DNxBCL2 50+/+	M	18	Spleen	VH1	IGHV1-74*04 F	VH102	D2	DSP2.4	JH4	89.9	9.7	-	0.35	UM	10/10	100	ASGYDYAMDY	10	3.56
TRAF2DNxBCL2 51+/+	M	18	Spleen	VH1	IGHV1-9*01 F	VHJ558.b9	D4	DQ52	JH4	96.9	2.4	-	0.7	UM	10/10	100	ARGNWDFYYAMDY	13	4.21
TRAF2DNxBCL2 55+/+ Parental	F	16	Spleen	VH1	IGHV1-74*04 F	VH102	D2	DSP2.4	JH4	90.3	9.7	-	0	UM	10/10	100	ASGYDYAMDY	10	3.56
			Blood	VH5	IGHV5-17*02 F	VH7183.a47.76	D2	DS	JH4	98.3	0.7	0.7	0.35	UM	5/10	50	AVYVIYDGYYGAMDY	15	3.56
				VH1	IGHV1-77*01 F	VHJ558.80.186	D1	DFL16.1e	JH2	94.8	3.8	-	1.4	UM	5/10	50	ARGGDY	6	5.88
TRAF2DNxBCL2 55+/+ F1	-	-	Node	VH1	IGHV1-77*01 F	VHJ558.80.186	D1	DFL16.1e	JH2	95.5	3.8	-	0.7	UM	9/10	90	ARGGDY	6	5.88
				VH1	IGHV1-9*01 F	VHJ558.b9	D2	DSP2.2	JH2	96.5	2.4	0.35	0.7	UM	1/10	10	ARGDYDGEFAY	11	4.03

**TRAF2DNxBCL2 65+/+**	**M**	**12**	**Spleen**	VH5	IGHV5-17*02 F	VH7183.a47.76	D3	DST4.3	JH2	98.3	1.0	0.7	0	UM	2/9	22,2	ALGAGYFDY	9	3.8
				VH1	IGHV1-69*02 F	VH124	D2	DSP2.9	JH1	92.7	2.1	0.35	4.9	M	2/9	22,2	ARGNDGSYWYFDV	13	4.21
				VH3	IGHV3-5*02 F	VH36-60.a5.112	D3	DST4	JH4	97.9	1.0	-	1.0	UM	2/9	22,2	ARIRGGAMDY	10	8.79
				VH1	IGHV1-9*01 F	VHJ558.b9	D1	DFL16.1	JH1	96.9	2.4	0.35	0.35	UM	1/9	11,1	ARHGSSYWYFDV	12	6.79
				VH5	IGHV5-17*02 F	VH7183.a47.76	D2	DSP2.9	JH4	97.9	1.0	0.35	0.7	UM	1/9	11,1	ARWLLRYYAMDY	12	8.54
				VH14	IGHV14-2*01 F	VHSM7.a2psi.88	D2	DSP2.9	JH4	96.5	3.1	-	0.35	UM	1/9	11,1	GRDDGYYYAMDY	12	3.93
			**Blood**	VH5	IGHV5-17*02 F	VH7183.a47.76	D3	DST4.3	JH2	98.3	1	0.35	0.35	UM	7/10	70	ALGAGYFDY	9	3.8
				VH14	IGHV14-2*02 P	VHSM7.a2psi.88	D2	DSP2.9	JH4	96.1	3.1	-	0.7	UM	3/10	30	GRDDGYYYAMDY	12	3.93
**TRAF2DNxBCL2 72+/+ Parental**	**F**	**15**	**Spleen**	VH14	IGHV14-2*02 P	VHSM7.a2psi.88	D2	DSP2.9	JH4	96.6	2.7	-	0.7	UM	4/14	29	GRDDGYYYAMDY	12	3.93
				VH1	IGHV1S130*01 [F]	VHJ558.f	D2	DSP2.2	JH2	97.9	1.7	-	0.35	UM	4/14	29	ARVRNWDFEDY	11	4.56
				VH5	IGHV5-17*02 F	VH7183.a46.76	D3	DST4.3	JH4	96.2	1.0	0.7	2.1	M	1/14	7	AREGPRRDYYAMDY	14	6.16
				VH3	IGHV3-5*02 F	VH36-60.a5.112	D3	DST4.3	JH4	98.3	1.0	-	0.7	UM	1/14	7	ARIRGGAMDY	10	8.79
				VH1	IGHV1-26*01 F	VHJ558.b29	Unknown	Unknown	JH2	93.1	unknown	.-	6.9	M*	1/14	7	ARRFDY	6	8.79
				VH14	IGHV14-2*01 F	VHSM7.a2psi.88	D2	DSP2.9	JH4	96.5	3.1	-	0.35	UM	1/14	7	GRDDGYYYAMDY	12	3.93
				VH14	IGHV14-3*02 F	VHSM7.a3.93	D1	DFL16.1	JH2	97.9	1.4	-	0.7	UM	1/14	7	ARSPYYYGSRALFDY	15	8.47
				VH1	IGHV1-67*01 P	VHJ558.b73	D3	DST4.2	JH3	95.1	3.8	-	1.0	UM	1/14	7	ARKGKVRGFAY	11	11.1
**TRAF2DNxBCL2 72+/+ F1**	**-**	**-**	**Spleen**	VH1	IGHV1S130*01 [F]	VHJ558.f	D2	DSP2.2	JH2	97.6	2.4	-	0	UM	7/10	70	ARVRNWDFEDY	11	4.56
				VH6	IGHV6-6*02 F	VHJ606.a6.127	D4	DQ52	JH2	99	1.0	-	0	UM	2/10	20	TSWDVNY	7	3.8
				VH1	IGHV1-74*04 F	VH102	D2	DSP2.4	JH4	89.6	9.7	-	0.7	UM	1/10	10	ASGYDYAMDY	10	3.56
**TRAF2DNxBCL2 74+/+ Parental**	**F**	**15**	**Spleen**	VH1	IGHV1S130*01 [F]	VHJ558.f	D1	DFL16.1	JH2	97.6	2.4	-	0	UM	6/10	60	ASGPDFDY	8	3.56
				VH1	IGHV1-9*01 F	VHJ558.b9	D2	DSP2.4	JH4	97.2	2.4	0.35	0	UM	3/10	30	ARGGYYGYDGDYYAMDY	17	3.93
				VH1	IGHV1S53*02 F	VH058	D2	DSP2.5	JH2	98.3	unknown	-	1.7	UM	1/10	10	YGNLDY	6	3.8
**TRAF2DNxBCL2 74+/+ F1**	**-**	**-**	**Spleen**	VH1	IGHV1-9*01 F	VHJ558.b9	D2	DSP2.4	JH4	96.9	2.4	-	0.7	UM	8/8	100	ARGGYYGYDGDYYAMDY	17	3.93

Supplementary Table 5. Most frequently used IGHV subgroups in *Traf2*DNx*BCL2*-tg$^{+/+}$ expanded CLL/SLL clones and in those from other representative mouse models of CLL

	CLL clones (% IGHV subgroup usage)			
**IGHV subgroup**	***Traf2*** **DNx*BCL2*-tg$^{+/+}$**	**E*μ-TCL-1*-tg**	***MDR$^{-/-}$*** **&** ***miR-15a/16-1$^{-/-}$***	***IgH-TEμ*****-tg**
**1**	53	50	36	39.5
**3**	5	-	-	-
**4**	-	9.5	-	-
**5**	26	-	-	-
**11**	-	10	21	39.5
**12**	-	10	14	-
**14**	16	-	-	-

The most commonly found IGHV subsets and their % of expression in CLL clones from the indicated CLL mouse models are shown. Data for the E*μ-TCL-1*-tg mice, the *MDR*$^{-/-}$ and *miR15a/16-1*$^{-/-}$ and the *IgH-TEμ* mice were from references 28, 32 and 45, respectively.

SUPPLEMENTARY TABLE 6. Characteristics of the HCDR3 (UM vs M) regions of the B cells from the *Traf2*DN*xBCL2*-tg mice of the different genotypes, including the expanded *Traf2*DN*xBCL2*-tg$^{+/+}$ CLL/SLL clones.

***Traf2* DNx*BCL2* genotype**	**SHM**	**%**	**length (average)**	**lenght (SHM)**	**IP (average)**	**IP (SHM)**
**-/-**	UM	46	11.92 ± 2.7	10.4 ± 2.4	5.52 ± 1.6	5.5 ± 1.7
	M	54		13.2 ± 2.3		5.7 ± 1.7
**+/-**	UM	78.6	10.71 ± 2.6	10.9 ± 2.4	5.98 ± 1.98	6.1 ± 2.2
	M	21.4		10 ± 3.4		6 ± 0.8
**-/+**	UM	60	12.11 ± 3.3	11.8 ± 3.4	5.83 ± 1.8	6.1 ± 1.5
	M	40		12.6 ± 3		5.4 ± 2.1
**+/+ (all)**	UM	80	11.44 ± 2.8	11.2 ± 2.7	5.37 ± 2.04	5.5 ± 2.2
	M	20		12.3 ± 3.1		5.1 ± 1.6
**+/+ CLL**	UM	85	11.6 ± 2.9	11.18 ± 2.9	4.54 ± 1.31	4.5 ± 1.4
	M	15		14 ± 1		4.6 ± 1.3

The percentage (%) of UM- and M-B cell clones, the HCDR3 length and isoelectric point (pI) (average ± SD) of the B cell clones isolated from the *Traf2*DN*xB CL2*-tg of the indicated genotypes and from the expanded *Traf2*DN*xBCL2*-tg$^{+/+}$ CLL/SLL clones distributed according their SHM status (UM or M) is shown. IGHV gene was considered M if it had >2 % variation from the GL sequence.

SUPPLEMENTARY TABLE 7. Identical HCDR3 sequences found in *Traf2*DNx*BCL2*-tg mice of the different genotypes

HCDR3	MOUSE ID	GENOTYPE	EXPRESSED TRANSGENES	FREQUENCY	IGHV GENE	IGHD GENE	IGHJ GENE	SHM
GRDDGYYYAMDY	13	+/+	*Traf2* DN; *BCL2*	**5/13**	VHSM7.a2psi.88	DSP2.9	JH4	UM
GRDDGYYYAMDY	65	+/+	*Traf2* DN; *BCL2*	**3/10**	VHSM7.a2psi.88	DSP2.9	JH4	UM
GRDDGYYYAMDY	72	+/+	*Traf2* DN; *BCL2*	**4/14**	VHSM7.a2psi.88	DSP2.9	JH4	UM
GRDDGYYYAMDY	72	+/+	*Traf2* DN; *BCL2*	1/14	VHSM7.a2psi.88	DSP2.9	JH4	UM
AREGPRRDYYAMDY	72	+/+	*Traf2* DN; *BCL2*	1/10	VH7183.a47.76	DST4.3	JH4	M
AREGPRRDYYAMDY	13	+/+	*Traf2* DN; *BCL2*	**3/13**	VH7183.a47.76	DST4.3	JH4	M
ARGDYDGEFAY	45	+/+	*Traf2* DN; *BCL2*	**8/10**	VHJ558.b9	DSP2.2	JH3	UM
ARGDYDGEFAY	55F1	+/+	*Traf2* DN; *BCL2*	1/10	VHJ558.b9	DSP2.2	JH2	UM
ARGGYYGYDGDYYAMDY	74	+/+	*Traf2* DN; *BCL2*	**3/10**	VHJ558.b9	DSP2.4	JH4	UM
ARGGYYGYDGDYYAMDY	74F1	+/+	*Traf2* DN; *BCL2*	**8/8**	VHJ558.b9	DSP2.4	JH4	UM
ARGGYYGYDGDYYAMDY	17	-/+	*BCL2*	1/15	VHJ558.b9	DSP2.4	JH4	M
ARIRGGAMDY	65	+/+	*Traf2* DN; *BCL2*	2/9	VH36-60.a5.112	DST4	JH4	UM
ARIRGGAMDY	13	+/+	*Traf2* DN; *BCL2*	1/13	VH36-60.a5.112	DST4	JH4	UM
ARIRGGAMDY	72	+/+	*Traf2* DN; *BCL2*	1/10	VH36-60.a5.112	DST4.3	JH4	UM
ARSPYYYGSRALFDY	13	+/+	*Traf2* DN; *BCL2*	1/13	VHSM7.a3.93	DFL16.1	JH2	UM
ARSPYYYGSRALFDY	72	+/+	*Traf2* DN; *BCL2*	1/14	VHSM7.a3.93	DFL16.1	JH2	UM
ARWLLRYYAMDY	13	+/+	*Traf2* DN; *BCL2*	1/13	VH7183	DSP2.9	JH4	UM
ARWLLRYYAMDY	65	+/+	*Traf2* DN; *BCL2*	1/9	VH7183	DSP2.9	JH4	UM
ASGYDYAMDY	50	+/+	*Traf2* DN; *BCL2*	**10/10**	V102	DSP2.4	JH4	UM
ASGYDYAMDY	55	+/+	*Traf2* DN; *BCL2*	**10/10**	V102	DSP2.4	JH4	UM
ASGYDYAMDY	72F1	+/+	*Traf2* DN; *BCL2*	1/10	VH102	DSP2.4	JH4	UM
ATYYGYDRVYYYAMDY	40	+/+	*Traf2* DN; *BCL2*	**9/10**	VH7183.a47.76	DSP2.6	JH4	UM
ATYYGYDRVYYYAMDY	14	-/-	Wild-type	1/10	VH7183.a47.76	DSP2.6	JH4	UM
ATYYGYDRVYYYAMDY	64	-/+	*BCL2*	2/10	VH7183.a47.76	DSP2.6	JH4	UM
ATYYGYDRVYYYAMDY	79	-/+	*BCL2*	1/12	VH7183.a30.50	DSP2.6	JH4	UM
MRYSSYDYDWYFDV	14	-/-	Wild-type	1/10	VH11.a2.92	DSP2.2	JH1	UM
MRYSSYDYDWYFDV	47	-/-	Wild-type	1/6	VH11.a2.92	DSP2.2	JH1	UM
MRYSSYDYDWYFDV	40	+/+	*Traf2* DN; *BCL2*	1/10	VH11.a2.92	DSP2.2	JH1	M
TSWDVNY	8	-/-	Wild-type	2/10	VHJ606.b5	DQ52	JH2	UM
TSWDVNY	24	+/-	*Traf2* DN	1/6	VHJ606.a6.127	DQ52	JH2	UM
TSWDVNY	58	+/-	*Traf2* DN	1/8	VHJ606.a6.127	DQ52	JH2	UM
TSWDVNY	72F1	+/+	*Traf2* DN; *BCL2*	2/10	VHJ606.a6.127	DQ52	JH2	UM

The sequence of identical HCDR3, the mouse ID and genotype of the mice where these HCDR3 were found, the expressed transgenes, the frequency of occurrence for each clone (highlighted in bold are those clones with a representation >25%), as well as the IGHV, IGHD and IGHJ genes producing this HCDR3 and the SHM status (M or UM) is shown. A IGHV sequence is considered M if shares < 98% homology with the corresponding germ line sequence.

SUPPLEMENTARY TABLE 8. Stereotyped HCDR3 sequences found in *Traf2*DNx*BCL2*-tg mice of the different genotypes and in other CLL mouse models

HCDR3	MOUSE ID	GENOTYPE	EXPRESSED TRANSGENES	CELL TYPE	IGHV subgroup	IGHV GENE	IGHD GENE	IGHJ GENE	SHM
MRYGYGYWYFDV	*Traf2* DNx*BCL2* #14	-/-	WT	*normal B*	VH11	VH11.a2.92	DSP2.4	JH1	UM
MRYGSSYWYFDV	*TCL1* -005	+	*TCL1*	*CLL*	VH11	VH11S1	DFL16.1	JH1	UM
MRY-SNYWYFDV	*TCL1* -006	+	*TCL1*	*CLL*	VH11	VH11S1	DSP2.x	JH1	UM
MRY-SNYWYFDV	*MDR* +/- #27	+/-	-	*CLL*	VH11	V235	DSP2.x	JH1	UM
MRY-SNYWYFDV	*IgH-TE* µ *	+	SV40	*CLL*	VH11	VH11-2	DSP2.5	JH1	UM
MRYG-NYWYFDV	*Traf2* DNx*BCL2* #14	-/-	WT	*normal B*	VH11	VH11.a2.92	DSP2.5	JH1	UM
MRYG-NYWYFDV	*Traf2* DNx*BCL2* #60	-/-	WT	*normal B*	VH11	VH11.a2.92	DSP2.5	JH1	UM
MRYG-NYWYFDV	*IgH-TEµ* **	+	SV40	*CLL*	VH11	VH11-2	DSP2.1	JH1	UM
MRYG-NYWYFDV	*MDR* -/- #138	-/-	-	*CLL*	VH11	V153	DSP2.1	JH1	UM
MRYGS-YWYFDV	*Traf2* DNx*BCL2* #60	-/-	WT	*normal B*	VH11	VH11.a2.92	DFL16.1	JH2	UM
MRYGG-YWYFDV	*IgH-TEµ* #E-32	+	SV40	*CLL*	VH11	VH11-2	DSP2.1	JH1	UM
TSWDVNY	*Traf2* DNx*BCL2* #8	-/-	Wild-type	*normal B*	VH6	VHJ606.b5	DQ52	JH2	UM
TSWDVNY	*Traf2* DNx*BCL2* #24	+/-	*Traf2* DN	*normal B*	VH6	VHJ606.a6.127	DQ52	JH2	UM
TSWDVNY	*Traf2* DNx*BCL2* #58	+/-	*Traf2* DN	*normal B*	VH6	VHJ606.a6.127	DQ52	JH2	UM
TSWDVNY	*Traf2* DNx*BCL2* #72F1	+/+	*Traf2* DN; *BCL2*	*normal B*	VH6	VHJ606.a6.127	DQ52	JH2	UM
TSWDVNY	*TRAF3* x*BCL2* #3	+/+	*Traf3* ; *BCL2*	*normal B*	VH6	VHJ606.a6.127	DQ52	JH2	UM
TSWDVGY	*TCL1* -019	+	*TCL1*	*CLL*	VH6	V6S1	DQ52	JH4	UM
AGDSTGYWYFDV	*Traf2* DNx*BCL2* # 58	+/-	*Traf2DN*	*normal B*	VH12	VH12.b3	DQ52	JH1	UM
AGDRTGYWYFDV	*TCL1* -002	+	*TCL1*	*CLL*	VH12	NC1-A7	DST4	JH1	UM
AGDRRGYWYFDV	*TCL1* -001	+	*TCL1*	*CLL*	VH12	NC1-A7	DST4	JH1	UM

The sequence of stereotyped HCDR3, the mouse model, mouse ID and genotype of the mice where these HCDR3 were found, the expressed transgenes and whether the cell expressing the HCDR3 is a CLL or a normal B cell clone is indicated. The IGHV subgroup and IGHV, IGHD and IGHJ genes producing this HCDR3 and the SHM status (M or UM) are also indicated. *HCDR3 found in mice E-01, E-06, C-ECD4-01, ECD4-04 and C-84. **HCDR3 found in mice E-07, E-20, EX-02, EX-03, ESG-C03, ESG-C11 and ESG-C13. Data for the E*µ*-*TCL-1*-tg mice, the *MDR*$^{-/-}$ and *miR15a/16-1*$^{-/-}$ and the *IgH-TEµ* mice were from references 28, 32 and 45, respectively.

Supplementary Table 9. Comparison of HCDR3 sequences with high homology to those found in *Traf2*DNx*BCL2*-tg$^{+/+}$B cell clones

a) anti-phosphatidylcholine HCDR3

*Traf2*DNx*BCL2*-tg$^{+/+}$	ARGGYYGYGYDYYAMDY
**consensus**	**ARG Y YGYDYYAMDY**
clone AAB07432.1	ARG--Y-YGYDYYAMDY

b) anti-cardiolipin HCDR3

*Traf2*DNx*BCL2*-tg$^{+/+}$	AR-GNDGSYWYFDV
**consensus**	**AR GNDG YWYFDV**
clone AAB71184.1	ARHGNDGYYWYFDV

c) anti-vaccinia protein A33 HCDR3

*Traf2*DNx*BCL2*-tg$^{+/+}$	ARIRGGAMDY
**consensus**	**AR GGAMDY**
clone AHB38924.1	ARQWGGAMDY

d) anti-*Bordetella pertussis* HCDR3

*Traf2*DNx*BCL2*-tg$^{+/+}$	ASPSY-DYPYYYAMDY
**consensus**	**A Y DYPYYYAMDY**
clone ABB13479.1	ARRVYDDYPYYYAMDY

e) anti-*Bordetella pertussis* HCDR3

*Traf2*DNx*BCL2*-tg$^{+/+}$	REGPRRDYYAMDY
**consensus**	**REG RR +YAMDY**
clone BAB87185.1	REGLRRAFYAMDY

f) anti-hepatitis c E33 protein HCDR3

*Traf2*DNx*BCL2*-tg$^{+/+}$	ARGDYDGEFAY
**consensus**	**AR DYDG FAY**
clone 4Q0X.H	ARDDYDGWFAY

Supplementary Figure 1. Expression of IGHV, IGHD and IGHJ subgroup usage in *Traf2*DNx*BCL2*-tg. Bars represent the number of B cell clones expressing each IGHV, IGHD and IGHJ subgroup found in mice representative of all different genotypic combinations ($^{-/-}$; $^{+/-}$; $^{-/+}$ and $^{+/+}$), including those found in the *Traf2*DNx*BCL2*-tg$^{+/+}$ expanded CLL/SLL clones.

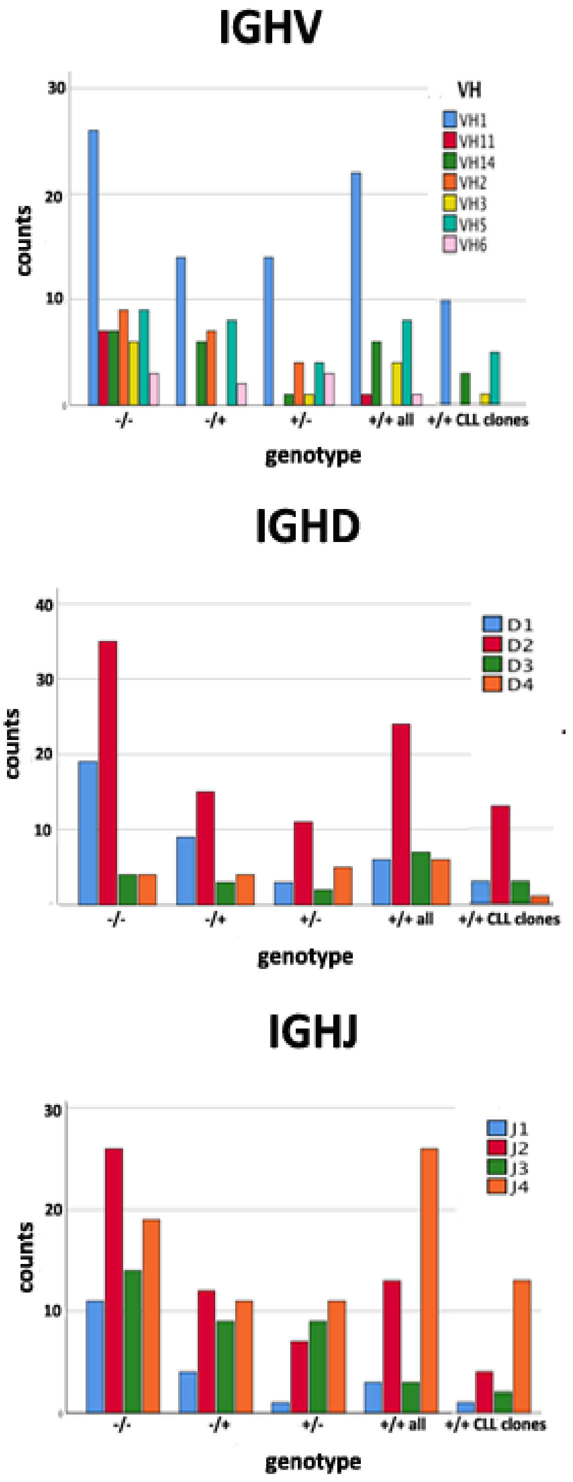

Supplementary Figure 2. Analysis of the IGHV, IGHD and IGHJ subgroup usage in the *Traf3xBCL2*-tg$^{+/+}$ mice. The IGHV, IGHD and IGHJ subgroup usage in all the B cell clones isolated from the *Traf3xBCL2*-tg$^{+/+}$ mice and of expanded B cell clones is shown. The percentage of the identified genes of each subgroup is indicated and represented in circle diagrams.

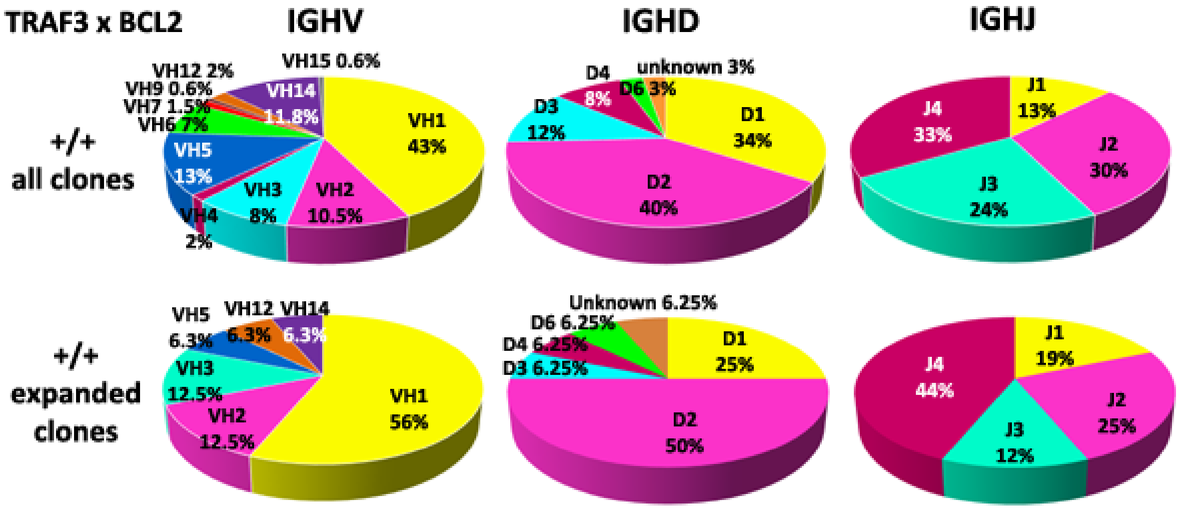

Supplementary Figure 3. Comparative sequence analysis of the IGHV regions of *Traf2*DNx*Bcl2*-tg B cell clones expressing the same IGHV gene and its IMGT reference V_H gene. The analysis was performed according to the criteria described in supplementary materials and methods. Probable strain-specific polymorphisms (SSPs) are highlighted in yellow and somatic hypermutations (SHMs) in blue. Uncertain spots are highlighted in green. The IMGT sequence of the referenced gene is shown in bold. A table summarizing the findings is shown. **A**. ClustalW sequence analysis of the IMGT-reference IGHV1-85*0 gene and the IGHV regions of this gene expressed by the *Traf2*DNx*Bcl2*-tg B cell clones indicated in the figure and for 2 mAb clones from FVB/N mice expressing IGHV1-85*0 available in the genebank (AF303863.1 and M36227.1). Probable strain-specific polymorphisms (SSPs) are highlighted in yellow and somatic hypermutations (SHMs) in blue. The IMGT sequence of the referenced gene is shown in bold. Similar analyses are shown in B for IGHV1-74*04 genes **C** for IGHV1-14*01 genes, **D** for IGHV1-80*01 genes, **E** for IGHV1-9*01 genes, **F** for IGHV7-3*02 genes, **G** for IGHV4-1*02 genes and **H** for IGHV3-6*01 genes

A

```
IGHV1-85*0      CAGGTTCAGCTGCAGCAGTCTGGACCTGAGCTGGTGAAGCCTGGGGCTTCAGTGAAGTTG
16+/+1          CAGGTTCAGCTGCAGCAGTCAGGAGCTGAACTGGTAAAGCCTGGGGCTTCAGTGAAGTTG
16+/+5          CAAGTTCAGCTGCAGGAGTCTGGAGCTGAACTGGTAAAGCCTGGGGCTTCAGTGAAGTTG
AF303863.1      CAGGTTCAGCTGCAGCAGTCTGGAGCTGAACTGGTAAAGCCTGGGGCTTCAGTGAAGTTG
M36227.1        CAGGTTCAGCTGCAGCAGTCTGGAGCTGAACTGGTAAAGCCTGGGGCTTCAGTGAAGTTG
                **.************ ****:*** ****.*****.************************

IGHV1-85*0      TCCTGCAAGGCTTCTGGCTACACCTTCACAAGCTACGATATAAACTGGGTGAAGCAGAGG
16+/+1          TCCTGCAAGGCTTCTGGCTACACCTTCACAAGCTATGATATAAACTGGGTGAGGCAGAGG
16+/+5          TCCTGCAAGGCTTCTGGCTACACCTTCACAAGCTATGATATAAACTGGGTGAGGCAGAGG
AF303863.1      TCCTGCAAGGCTTCTGGCTACACCTTCACAAGCTATGATATAAACTGGGTGAGGCAGAGG
M36227.1        TCCTGCAAGGCTTCTGGCTACACCTTCACAAGCTATGATATAAACTGGGTGAGGCAGAGG
                *********************************** ****************.*******

IGHV1-85*0      CCTGGACAGGGACTTGAGTGGATTGGATGGATTTATCCTAGAGATGGTAGTACTAAGTAC
16+/+1          CCTGAACAGGGACTTGAGTGGATTGGATGGATTTTTCCTGGAGATGGTAGTACTAAGTAC
16+/+5          CCTGAACAGGGACTTGAGTGGATTGGATGGATTTTTCCTGGAGATGGTAGTACTAAGTAC
AF303863.1      CCTGAACAGGGACTTGAGTGGATTGGATGGATTTTTCCTGGAGATGGTAGTACTAAGTAC
M36227.1        CCTGAACAGGGACTTGAGTGGATTGGATGGATTTTTCCTGGAGATGGTAGTACTAAGTAC
                ****.*****************************:****.********************

IGHV1-85*0      AATGAGAAGTTCAAGGGCAAGGCCACATTGACTGTAGACACATCCTCCAGCACAGCGTAC
16+/+1          AATGAGAAGTTCAAGGGCAAGGCCACACTGACTACAGACAAATCCTCCAGCACAGCCTAC
16+/+5          AATGAGAAGTTCAAGGGCAAGGCCACACTGACTACAGACAAATCCTCCAGCACAGCCTAC
AF303863.1      AATGAGAAGTTCAAGGGCAAGGCCACACTGACTACAGACAAATCCTCCAGCACAGCCTAC
M36227.1        AATGAGAAGTTCAAGGGCAAGGCCACACTGACTACAGACAAATCCTCCAGCACAGCCTAC
                *************************** *****. *****.*************** ***

IGHV1-85*0      ATGGAGCTCCACAGCCTGACATCTGAGGACTCTGCGGTCTATTTCTGTGCAA
16+/+1          ATGCAGCTCAGCAGGCTGACATCTGAGGACTCTGCTGTCTATTTCTGTGCAA
16+/+5          ATGCAGCTCAGCAGGCTGACATCTGAGGACTCTGCTGTCTATTTCTGTGCAA
AF303863.1      ATGCAGCTCAGCAGGCTGACATCTGAGGACTCTGCTGTCTATTTCTGTGCAA
M36227.1        ATGCAGCTCAGCAGGCTGACATCTGAGGACTCTGCTGTCTATTTCTGTGCAA
                *** *****..*** ******************** ****************
```

	SSP	%	SHM	%	IMGT
16+/+1	18	6.3	1	0.35	93.4
16+/+5	18	6.3	2	0.7	93

B

```
IGHV1-74*04    CATGTCCAACTGCAGCAGCCTGGGGCTGAGCTGGTGAAGCCTGGGGCTTCAGTGAAGGTG
55+/+#1        CAAGTCAAGCTGCAGCAGTCTGGGCCTCAGCTGGTTAGGCCTGGGGCTTCAGTGAAGATA
72F1+/+#3      CAAGTTCAGCTGCAGGAGTCTGGGCCTCAGCTGGTTAGGCCTGGGGCTTCAGTGAAGATA
58+/-#7        CAAGTCAAGCTGCAGGAGTCAGGGCCTCAGCTGGTTAGGCCTGGGGCTTCAGTGAAGATA
50+/+#1        CAAGTTCAGCTGCAGCAGTCTGGGCCTCAGCTGGTTAGGCCTGGGGCTTCAGTGAAGATA
79-/+#10       CAAGTTAAGCTGCAGCAGTCTGGGGCTGAGCTGGTGAGGCCTGGAGCTTCAGTGAAGCTG
71-/-#4        CAAGTTAAGCTGCAGCAGTCAGGGGCTGAGCTGGTGAGGCCTGGAGCTTCAGTGAAGCTG
               **:** .*.****** ** *:*** ** ******* *.******.************ *.

IGHV1-74*04    TCCTGCAAGGCTTCTGGCTACACCTTCACCAGCTACTGGATGCACTGGGTGAAGCAGAGG
55+/+#1        TCCTGCAAGGCTTCTGGTTACTCATTCACCAGCTACTGGATGCACTGGGTGAAGCAGAGG
72F1+/+#3      TCCTGCAAGGCTTCTGGTTACTCATTCACCAGCTACTGGATGCACTGGGTGAAGCAGAGG
58+/-#7        TCCTGCAAGGCTTCTGGTTACTCATTCACCAGCTACTGGATGCACTGAGTGAAGCAGAGG
50+/+#1        TCCTGCAAGGCTTCTGGTTACTCATTCACCAGCTACTGGATGCACTGGGTGAAGCAGAGG
79-/+#10       TCCTGCAAGGCCTCTGGCTACTCCTTCACCAGCTACTGGATGAACTGGGTGAAGCAGAGG
71-/-#4        TCCTGCAAGGCTTCTGGCTACTCCTTCACCAGCTACTGGATGAACTGGGTGAAGCAGAGG
               *********** ***** ***:*.*****************.****.************

IGHV1-74*04    CCTGGCCAAGGCCTTGAGTGGATTGGAAGGATTCATCCTTCTGATAGTGATACTAACTAC
55+/+#1        CCTGGACAAGGTCTTGAGTGGATTGGCATGATTGATCCTTCCGATAGTGAAACTAGGTTA
72F1+/+#3      CCTGGACAAGGTCTTGAGTGGATTGGCATGATTGATCCTTCCGATAGTGAAACTAGGTTA
58+/-#7        CCTGGACAAGGTCTTGAGTGGATTGGCATGATTGATCCTTCCGATAGTGAAACTAGGTTA
50+/+#1        CCTGGACAAGGTCTTGAGTGGATTGGCATGATTGATCCTTCCGATAGTGAAACTAGGTTA
79-/+#10       CCTGGACAAGGCCTTGAGTGGATTGGCATGATTCATCCTTCCGAAAGTGAAACTAGGTTA
71-/-#4        CCTGGACAAGGCCTTGAGTGGATTGGCATGATTCATCCTTCCGATAGTGAAACTAGGTTA
               *****.***** **************.* **** ******* **:*****:****. *:.

IGHV1-74*04    AATCAAAAGTTCAAGGGCAAGGCCACATTGACTGTAGACAAATCCTCCAGCACAGCCTAC
55+/+#1        AATCAGAAGTTCAAGGACAAGGCCACATTGACTGTAGACAAATCCTCCAGCACAGCCTAC
72F1+/+#3      AATCAGAAGTTCAGGGACAAGGCCACATTGACTGTAGACAAATCCTCCAGCACAGCCTAC
58+/-#7        AATCAGAAGTTCAAGGACAAGGCCACATTGACTGTAGACAAATCCTCCAGCACAGCCTAC
50+/+#1        AATCAGAAGTTCAAGGACAAGGCCACATTGACTGTAGACGAATCCTCCAGCACAGCCTAC
79-/+#10       AATCAGAACTTCAAGGACAAGGCCACATTGACTGTAGACAAATCCTCCAGCATAGCCTAC
71-/-#4        AATCAGAAGTTCAAGGACAAGGCCACATTGACTGTAGACAAATCCTCCAGCACAGCCTAC
               *****.** ****.**.*********************.************ *******

IGHV1-74*04    ATGCAGCTCAGCAGCCTGACATCTGAGGACTCTGCGGTCTATTACTGT
55+/+#1        ATGCAACTCAGCAGCCCGACATCTGAGGACTCTGCGGTCTATTACTGT
72F1+/+#3      ATGCAACTCAGCAGCCCGACATCTGAGGACTCTGCGGTCTATTACTGT
58+/-#7        ATGCAACTCAGCAGCCCGACATCTGAGGACTCTGTGGTCTATTACTGT
50+/+#1        ATGCAACTCAGCAGCCCGACATCTGAGGACTCTGCGGTCTATTACTGT
79-/+#10       ATGAAACTCAGCAGCCCGACATCTGAGGACTCTGCGGTCTATTACTGT
71-/-#4        ATGCAACTCAGCAGCCCGACATCTGAGGACTCTGCGGTCTATTACTGT
               ***.*.********** ***************** *************
```

	SSP	%	SHM	%	IMGT
55+/+#1	27	9.4	0	0	90.3
72F1+/+#3	27	9.4	2	0.7	89.6
58+/-#7	27	9.4	4	1.4	88.9
50+/+#1	27	9.4	1	0.35	89.9
79-/+#10	19	6.6	9	3.1	90.3
71-/-#4	20	6.9	4	1.4	91.7

C

```
IGHV1-14*01                    GAGTTCCAGCTGCAGCAGTCTGGACCTGAGCTGGTAAAGCCTGGGGCTTC
EPS/BCL2_3_Parental_10         CAAGTTAAGCTGCAGGAGTCAGGACCTGAGCTGGTAAAGCCTGGGGCTTC
35-/-                          CAAGTTAAGCTGGAGGAGTCTGGACCTGAGCTGGTAAAGCCTGGGGCTTC
64-/+                          CAAGTTAAGCTGCAGGAGTCAGGACCTGAGCTGGTAAAGCCTGGGGCTTC
EPS/BCL2_232_11                CAAGTTAAGCTGCAGCAGTCAGGACCTGAGCTGGTAAAGCCTGGAGCTTC
EPS/BCL2_232_                  CAGGTTCAGCTGGAGCAGTCTGGACCTGAGCTGGTAAAGCCTGGGGCTTC
EPS/BCL2_232_7                 CGGGATTAGCTGCAGGAGTCTGGACCTGAGCTGGTAAAGCCTGGGGCTTC
                                ..  :  ***** ** ****:***********************.*****

IGHV1-14*01                    AGTGAAGATGTCCTGCAAGGCTTCTGGATACACATTCACTAGCTATGTTA
EPS/BCL2_3_Parental_10         AGTGAAGATGTCCTGCAAGGCTTCTGGATACACATTCACTAGCTATGTTA
35-/-                          AGTGAAGATGTCCTGCAAGGCTTCTGGATACACATTCACTAGCTATGTTA
64-/+                          AGTGAAGATGTCCTGCAAGGCTTCTGGATACACATTCACTAGCTATGTTA
EPS/BCL2_232_11                AGTGAAGATGTCCTGCAAGGCTTCTGGATACACATTCACTAGCTATGTTA
EPS/BCL2_232_                  AGTGAAGATGTCCTGCAAGGCTTCTGGATACACATTCACTAGCTATGTTA
EPS/BCL2_232_7                 AGTGAAGATGTCCTGCAAGGCTTCTGGATACACATTCACTAGCTATGTTA
                               **************************************************

IGHV1-14*01                    TGCACTGGGTGAAGCAGAAGCCTGGGCAGGGCCTTGAGTGGATTGGATAT
EPS/BCL2_3_Parental_10         TGCACTGGGTGAAGCAGAAGCCTGGGCAGGGCCTTGAGTGGATTGGATAT
35-/-                          TGCACTGGGTGAAGCAGAAGCCTGGGCAGGGCCTTGAGTGGATTGGATAC
64-/+                          TGCACTGGGTGAAGCAGAAGCCTGGGCAGGGCCTTGAGTGGATGGGATAT
EPS/BCL2_232_11                TGCACTAGGTGAAGCAGAAGCCTGGGCAGGGCCTTGAGTGGATTGGATAT
EPS/BCL2_232_                  TGCACTAGGTGAAGCAGAAGCCTGGGCAGGGCCTTGAGTGGATTGGATAT
EPS/BCL2_232_7                 TGCACTAGGTGAAGCAGAAGCCTGGGCAGGGCCTTGAGTGGATTGGATAT
                               ******.************************************ *****

IGHV1-14*01                    ATTTATCCTTACAATGATGGTACTAAGTACAATGAGAAGTTCAAAGGCAA
EPS/BCL2_3_Parental_10         ATTAATCCTTACAATGATGGTACTAAGTACAATGAGAAGTTCAAAGGCAA
35-/-                          ATTAATCCTAGCACTGGTTATACTGAGTACAATCAGAAGTTCAAGGACAA
64-/+                          ATTAATCCTTACAATGATGGTACTAAGTACAATGAGAAGTTCAAAGGCAA
EPS/BCL2_232_11                ATTAATCCTTACAATGATGGTACTAAGTACAATGAGAAGTTCAAAGGCAA
EPS/BCL2_232_                  ATTAATCCTTACAATGATGGTACTAAGTACAATGAGAAGTTCAAAGGCAA
EPS/BCL2_232_7                 ATTAATCCTTACAATGATGGTACTAAGTACAATGAGAAGTTCAAAGGCAA
                               ***:*****:.**.**.* .****.******** **********.*.***

IGHV1-14*01                    GGCCACACTGACTTCAGACAAATCCTCCAGCACAGCCTACATGGAGCTCA
EPS/BCL2_3_Parental_10         GGCCACACTGACTTCAGACAAATCCTCCAGCACAGCCTACATGGAGCTCA
35-/-                          GGCCACATTGACTGCAGACAAATCCTCCAGCACAGCCTACATGCAACTGG
64-/+                          GGCCACACTGACTTCAGACAAATCCTCCAGCACAGCCTACATGGAGCTCA
EPS/BCL2_232_11                GGCCACACTGACTTCAGACAAATCCTCCAGCACAGCCTACATGGAGCTCA
EPS/BCL2_232_                  GGCCACACTGACTTCAGACAAATCCTCCAGCACAGCCTACATGGAGCTCA
EPS/BCL2_232_7                 GGCCACACTGACTTCAGACAAATCCTCCAGCACAGCCTACATGGAGCTCA
                               ******* ***** ***************************** *.** .

IGHV1-14*01                    GCAGCCTGACCTCTGAGGACTCTGCGGTCTATTACTGTGCAA
EPS/BCL2_3_Parental_10         GCAGCCTGACCTCTGAGGACTCTGCGGTCTATTACTGTGCAA
35-/-                          GCAGCCTGACATCTGAGGACTCTGCAGTCTATTACTGTGCAA
64-/+                          GCAGCCTGACCTCTGAGGACTCTGCGGTCTATTACTGTGCAA
EPS/BCL2_232_11                GCAGCCTGACCTCTGAGGACTCTGCGGTCTATTACTGTGCAA
EPS/BCL2_232_                  GCAGCCTGACCTCTGAGGACTCTGCGGTCTATTACTGTGCAA
EPS/BCL2_232_7                 GCAGCCTGACCTCTGAGGACTCTGCGGTCTATTACTGTGCAA
                               **********.***************.***************
```

	SSP	%	SHM	%	IMGT
35-/- #2	7	2.4	20	6.9	90.6
64-/+ #5	8	2.7	1	0.35	96.9

D

```
IGHV1-80*01      CAGGTTCAGCTGCAGCAGTCTGGGGCTGAGCTGGTGAAGCCTGGGGCCTCAGTGAAGATT
17-/+#11         CAAGTTAAGCTGCAGGAGTCTGGGGCTGAACTGGTGAGGCCTGGGTCCTCAGTGAAGATT
29+/+#1          CAGGACAAGCTGGAGGAGTCTGGGGATGAGCTGGTGAGGCCTGGGTCCTCAGTGAAGATT
58+/-#1          CAGGTCAAGCTGGAGCAGTCAGGGGCTGAGCTGGTGAGGCCTGGGTCCTCAGTGAAGATT
8-/-#9           CAAGTTAAGCTGGAGCAGTCAGGGGCTGAACTGGTGAGGCCTGGGTCCTCAGTGCAGATT
                 **.*: .***** ** ****:****.***.*******.******* ********.*****

IGHV1-80*01      TCCTGCAAAGCTTCTGGCTACGCATTCAGTAGCTACTGGATGAACTGGGTGAAGCAGAGG
17-/+#11         TCCTGCAAGGCTTCTGGCTATGCATTCAGTAGCTACTGGATGAACTGGGTGAAGCAGAGG
29+/+#1          TCCTGCAAGGCTTCTGGCTATGCATACAGTAGCTACTGGATGAACTGGGTGAAGCAGAGG
58+/-#1          TCCTGCAAGGCTTCTGGCTACGCATTCAGTAGCTCCTGGATAAACTGGGTGAAGCAGAGG
8-/-#9           TCCTGCAAGACTTCTGGCTACTCATTCGTTAGTTACTGGATAAACTGGGTGAAACAGAGG
                 ********..**********  ***:*. *** *.******.***********.******

IGHV1-80*01      CCTGGAAAGGGTCTTGAGTGGATTGGACAGATTTATCCTGGAGATGGTGATACTAACTAC
17-/+#11         CCTGGACAGGGTCTTGAGTGGATTGGACAGATTTATCCTGGAGATGATGATACTAACTAC
29+/+#1          CCTGGACAGGGTCTTGAGTGGATTGGACAGATTTATCCTGGAGATGGTGATACTAACTAC
58+/-#1          CCTGGACAGGGTCTTGAGTGGATTGGACAGATTTATCCTGGAGATGGTGATACTAACTAC
8-/-#9           CCCGGACAGGGTCTTGAATGGATTGGACAGATTTATCCTGGAGATGATGATACTAACTAC
                 ** ***.**********.*****************************.************

IGHV1-80*01      AACGGAAAGTTCAAGGGCAAGGCCACACTGACTGCAGACAAATCCTCCAGCACAGCCTAC
17-/+#11         ACTGAAAAATTCAAGGGTAAAGCCACACTGACTGCAGACAAATCCTCCAGCACAGCCTAC
29+/+#1          AATGGAAAGTTCAAGGGTAAAGCCACACTGACTGCAGACAAATCCTCCAGCACAGCCTAC
58+/-#1          AATGGAAAGTTCAAGGGTAAAGCCACACTGACTGCAGACAAATCCTCCAGCACAGCCTAC
8-/-#9           AATGGAATGTTCAAAAATAAAGCCACACTGACTGCAGACAAATCCTCCAATACAGTCTAC
                 *. *.**:.*****... **.****************************. **** ****

IGHV1-80*01      ATGCAGCTCAGCAGCCTGACCTCTGAGGACTCTGCGGTCTATTTCTGTGCAAGA
17-/+#11         ATGCAGCTCAGCAGCCTAACATCTGAGGACTCTGCGGTCTATTTCTGTGCAAGA
29+/+#1          ATGCAGCTCAGCAGCCTAACATCTGAGGACTCTGCGGTCTATTTCTGTGCAAGA
58+/-#1          ATGCAGCTCAGCAGCCTAACATCTGAGGACTCTGCGGTCTATTTCTGTGCAAGA
8-/-#9           TTGCAACTCAGCAGCCTGACATCTGGGGACTCTGCGGTCTACTTCTGTGCAAGA
                 :****.***********.**.****.*************** ***********
```

	SSP	%	SHM	%	IMGT
17-/+#11	10	3.5	8	2.7	93.8
29+/+#1	10	3.5	6	2.1	94.4
58+/-#1	11	3.8	4	1.4	94.8
8-/-#9	10	3.5	25	8.7	87.8

E

```
IGHV1-9*01      CAGGTTCAGCTGCAGCAGTCTGGAGCTG--AGCTGATGAAGCCTGGGGCCTCAGTGAAGC
17-/+#1         CTG--CAGGCGGCCGCACTAGTGATTGTGCAGCTGGTGGAGTCTGGGGCCTCAGTGAAGA
79-/+#4         GAAGTTAAGCTGCAGGAGTCTGGAGCTG--AGCTGATGAAGCCTGGGGCCTCAGTGAAGA
55F1+/+#2       CAGGTCAAGCTGCAGCAGTCAGGAGCTG--AGCTGATGAAGCCTGGGGCCTCAGTGAAGA
51+/+#1         CAAGTCAAGCTGCAGCAGTCTGGAGCTG--AGCTGATGAAGCCTGGGGCCTCAGTGAAGA
14-/-#10        CAAGTCCAGCTGCAGGAGTCTGGAGTTG--AACTGATGAAGCCTGGGGCCTCAGTGAAGA
74F1+/+#1       CAGGTGAAGCTGGAGCAGTCTGGAGCTG--AGCTGATGAAGCCTGGGGCCTCAGTGAAGA
68+/-#5         CAAGTTAAGCTGCAGCAGTCTGGAGCTG--AGCTGATGAAGCCTGGGGCCTCAGTGAAGA
74+/+#2         CAGGTTAAGCTGCAGCAGTCAGGAGCTG--AGCTGATGAAGCCTGGGGCCTCAGTGAAGA
65+/+#4         CAGGTTAAGCTGCAGCAGTCAGGAGCTG--AGCTGATGAAGCCTGGGGCCTCAGTGAAGA
45+/+#1         CAGGTTAAGCTGGAGGAGTCAGGAGCTG--AGCTGATGAAGCCTGGGGCCTCAGTGAAGA
58+/-#2         CAGGTTAAGCTGGAGCAGTCAGGAGATG--ATCTGGTAAAGCCTGGGGCCTCAGTGAAGC
                 :.   ..** * .* * *.  **      * ***.*..** *****************.

IGHV1-9*01      TTTCCTGCAAGGCTACTGGCTACACATTCACTGGCTACTGGATAGAGTGGGTAAAGCAGA
17-/+#1         TATCCTGCAAGGCTACTGGCTACACACTCAGTAGCTACTGGATAGAGTGGGTAAAGCAGA
79-/+#4         TATCCTGCAAGGCTACTGGCTACACATTCAGTAGCTACTGGATAGAGTGGGTAAAGCAGA
55F1+/+#2       TATCCTGCAAGGCTACTGGCTACACATTCAGTAGCTACTGGATAGAGTGGGTAAAGCAGA
51+/+#1         TATCCTGCAAGGCTACTGGCTACACATTCAGTAGCTACTGGATAGAGTGGGTAAAGCAGA
14-/-#10        TCTCCTGCAAGGCTTCTGGCTACAAATTCAGTAATTACTGGATAGAGTGGATAAAAATGA
74F1+/+#1       TATCCTGCAAGGCTACTGGCTACACATTCAGTAGCTACTGGATAGAGTGGGTAAAGCAGA
68+/-#5         TATCCTGCAAGGCTACTGGCTACACATTCAGTAGCTACTGGATAGGGTGGGTAAAGCAGA
74+/+#2         TATCCTGCAAGGCTACTGGCTACACATTCAGTAGCTACTGGATAGAGTGGGTAAAGCAGA
65+/+#4         TATCCTGCAAGGCTACTGGCTACACATTCAGTAGCTACTGGATAGAGTGGGTAAAGCAGA
45+/+#1         TATCCTGCAAGGCTACTGGCTACACATTCAGTAGCTACTGGATAGAGTGGGTAAAGCAGA
58+/-#2         TGTCCTGCAAGGCTTCTGGCTACACCTTCACCAGCTACTGGATTAACTGGATAAAACAGA
                * ************:*********.. ***  .. ********:.. ***.****..:**

IGHV1-9*01      GGCCTGGACATGGCCTTGAGTGGATTGGAGAGATTTTACCTGGAAGTGGTAGTACTAACT
17-/+#1         GGCCTGGACATGGCCTTGAGTGGATTGGAGAGATTTTACCTGGAAGTGGTAGTACTAACT
79-/+#4         GGCCTGGACATGGCCTTGAGTGGATTGGAGAGATTTTACCTGGAAGTGGTAGTACTAACT
55F1+/+#2       GGCCTGGACATGGCCTTGAGTGGATTGGAGGGATTTTACCTGGAAGTGGTAGTACTAACT
51+/+#1         GGCCTGGACATGGCCTTGAGTGGATTGGAGAGATTTTACCTGGAAGTGGTAGTACTAACT
14-/-#10        GGCCCGGACACGGCCTTGAGTGGATTGGGGAGATTTTACCTGGAAGTAAAAGTTCCAAGT
74F1+/+#1       GGCCTGGACATGGCCTTGAGTGGATTGGAGAGATTTTACCTGGAAGTGGTAGTACTAACT
68+/-#5         GGCCTGGACATGGCCTTGAGTGGATTGGAGAGATTTTACCTGGAAGTGGTAGTACTAACT
74+/+#2         GGCCTGGACATGGCCTTGAGTGGATTGGAGAGATTTTACCTGGAAGTGGTAGTACTAACT
65+/+#4         GGCCTGGACATGGCCTTGAGTGGATTGGAGTGATTTTACCTGGAAGTGGTAGTACTAACT
45+/+#1         GGCCTGGACATGGCCTTGAGTGGATTGGAGAGATTTTACCTGGAAGTGGTAGTACTAACT
58+/-#2         GGCCTGGACAGGGCCTTGAGTGGATAGGACGTATTGCTCCTGGAAGTGGTAGTACTAACT
                **** ***** **************:**.   ***  :*********..:***:* ** *

IGHV1-9*01      ACAATGAGAAGTTCAAGGGCAAGGCCACATTCACTGCAGATACATCCTCCAACACAGCCT
17-/+#1         ACAATGAGAAGTTCAAGGGCAAGGCCACATTCACTGCAGATACATCCACCAACACAGCCT
79-/+#4         ACAATGAGAAGTTCAAGGGCAAGGCCACATTCACTGCAGATACATCCTCCAACACAGCCT
55F1+/+#2       ACAATGAGAAGTTCAAGGGCAAGGCCACATTCACTGCAGATACATCCTCCAACACAGCCT
51+/+#1         ACAATGAGAAGTTCAAGGGCAAGGCCACATTCACTGCAGATACATCCTCCAACACAGCCT
14-/-#10        ACAATGAGAAGTTCAGGGGCAAGGCCACATTCACTGCAGATTCATCCTCCGTCACAGCCT
74F1+/+#1       ACAATGAGAAGTTCAAGGGCAAGGCCACATTCACTGCAGATACATCCTCCAACACAGCCT
68+/-#5         ACAATGAGAAGTTCAAGGGCAAGGCCACATTCACTGCAGATACATCCTCCAACACAGCCT
74+/+#2         ACAATGAGAAGTTCAAGGGCAAGGCCACATTCACTGCAGATACATCCTCCAACACAGCCT
65+/+#4         ACAATGAGAAGTTCAAGGGCAAGGCCACATTCACTGCAGATACATCCTCCAACACAGCCT
45+/+#1         ACAATGAGAAGTTCAAGGGCAAGGCCACATTCACTGCAGATACATCCTCCAACACAGCCT
58+/-#2         ACAATGAGAAGTTCAAGGGCAAGGCCACATTCACTGCAGATACATCCTCCAACACAGCCT
                ***************.*************************:*****:**.:********
```

```
IGHV1-9*01     ACATGCAACTCAGCAGCCTGACAACTGAGGACTCTGCCATCTATTACTGTGCAAGA
17-/+#1        ACATGCAACTCAGCAGCCTGACATCTGAGGACTCTGCCGTCTATTACTGTGCAAGA
79-/+#4        ACATGCAACTCAGCAGCCTGACATCTGAGGACTCTGCCGTCTATTACTGTGCAAGA
55F1+/+#2      ACATGCAACTCAGCAGCCTGACATCTGAGGACTCTGCCGTCTATTACTGTGCAAGA
51+/+#1        ACATGCAACTCAGCAGCCTGACATCTGAGGACTCTGCCGTCTATTACTGTGCAAG-
14-/-#10       ACATGGAACTCAGCAGCCTGACATCTGAGGACTCTGCCGTATATTACTGTGCAAGA
74F1+/+#1      ACATGCAACTCAGCAGCCTGACATCTGAGGACTCTGCCGTCTATTACTGTGCAAGA
68+/-#5        ACATGCAACTCAGCAGCCTGACATCTGAGGACTCTGCCGTCTATTACTGTGCAAG-
74+/+#2        ACATGCAACTCAGCAGCCTGACATCTGAGGACTCTGCCGTCTATTACTGTGCAAGA
65+/+#4        ACATGCAACTCAGCAGCCTGACATCTGAGGACTCTGCCGTCTATTACTGTGCAAGA
45+/+#1        ACATGCAACTCAGCAGCCTGACATCTGAGGACTCTGCCGTCTATTACTGTGCAAGA
58+/-#2        ACATGCAACTCAGCAGCCTGACATCTGAGGACTCTGCCGTCTATTACTGTGCAAGA
               ***** *****************:**************.*.************** 
```

	SSP	%	Uncertain	%	SHM	%	IMGT
17-/+#1	5	1.74			19	6.6	91.7
79-/+#4	7	2.4			3	1	96.5
55F1+/+#2	7	2.4	1	0.35	2	0.7	96.5
51+/+#1	7	2.4			2	0.7	96.9
14-/-#10	5	1.74			28	9.7	88.5
4F1+/+#1	7	2.4			2	0.7	96.9
8+/-#5	7	2.4			2	0.7	96.9
74+/+#2	7	2.4	1	0.35	0	0	97.2
5+/+#4	7	2.4	1	0.35	1	0.35	96.9
45+/+#1	7	2.4	1	0.35	2	0.7	96.5
58+/-#2	4	1.4	1	0.35	22	7.6	90.6

F

```
IGHV7-3*02     GAGGTGAAGCTGGTGGAGTCTGGAGGAGGCTTGGTACAGCCTGGGGGTTCTCTGAGACTC
37-/-#9        CAAGTCAAGCTGCAGCAGTCTGGAGGAGGCTTGGTAAAGCCTGGGGGTTCTCTGAGACTC
29+/+#2        CAAGTTAAGCTGCAGGAGTCAGGAGGAGGCTTGGTACAGCCTGGGGGTTCTCTGAGACTC
64-/+#7        CAAGTTAAGCTGCAGCAGTCTGGAGGAGGCTTGGTACAGCCTGGGGGTTCTCTGAGACTC
64-/+#6        GAAGTTAAGCTGCAGGAGTCAGGANGAGGCTTGGTACAGTCTGGGGGTTCTCTGAGACTC
79-/+#5        GAGGTTAAGCTGGAGGAGTCAGGAGGAGGCTTGGTACAGCCTGGAGGTTCTCTGAGACTC
EPS/BCL2_39_1  CAGGTTAAGCTGCAGGAGTCAGGAGGAGGCTTGGTACAGCCTGGGGGTTCTCTGAGACTC
EPS/BCL2_14_3  CAGGTCAAGCTGCAGGAGTCAGGAGGAGGCTTGGTACAGCCTGGGGCTTCTCTGAGACTC
                *.** ****** :*  ****:***.***********.**  ****.***************

IGHV7-3*02     TCCTGTGCAACTTCTGGGTTCACCTTCACTGATTACTACATGAGCTGGGTCCGCCAGCCT
37-/-#9        TCCTGTGCAACTTCTGGGTTCACCTTCAGTGATTACTACTTGAACTGGGTCCGCCAGCCT
29+/+#2        TCCTGTGCAACTTCTGGGTTCACCTTCACTGATTACTACATGAGCTGGGTCCGCCAGCCT
64-/+#7        TCCTGTGCAACTTCTGGGTTCACCTTCACTGATTACTACATGAGCTGGGTCCGCCAGCCT
64-/+#6        TCCTGTGCGACTTCTGGGTTCACCTTCACTGATTACTACATGAACTGGGTCCGCCAGCCT
79-/+#5        TCCTGTGCAACTTCTGGGTTCACCTTCACTGATTACTACATGAGCTGGGTCCGCCAGCCT
EPS/BCL2_39_1  TCCTGTGCAACTTCTGGGTTCACCTTCACTGATTACTACATGAGCTGGGTCCGCCAGCCT
EPS/BCL2_14_3  TCCTGTGCAACTTCTGGGTTCACCTTCACTGATTACTACATGAGCTGGGTCCGCCAGCCT
               ********.******************* **********:***.****************

IGHV7-3*02     CCAGGAAAGGCACTTGAGTGGTTGGGTTTTATTAGAAACAAAGCTAATGGTTACACAACA
37-/-#9        CCAGGAAGGGCATTTGAGTGGTTGGGTTTCATTAGAAACAAAGCTAATGGTTACACAACA
29+/+#2        CCAGGAAAGGCACTTGAGTGGTTGGGTTTTATTAGAAACAAAGCTAATGGTTACACAACA
64-/+#7        CCAGGAAAGGCACTTGAGTGGTTGGGTTTTATTAGAAACAAAGCTAATGGTTACACAACA
64-/+#6        CCAGGAAAGGCACTTGAGTGGTTGGGTTTTATTAGAAACAATGCCAATGGTTTCACAACA
79-/+#5        CCAGGAAAGGCACTTGAGTGGTTGGGTTTTATTAGAAACAAAGCTAATGGTTACACAACA
EPS/BCL2_39_1  CAGGTTAAGCTGCAGGAGTCAGGAGGAGGCTTGGTACAGCCTGGGGGTTCTCTGAGACTC
EPS/BCL2_14_3  CCAGGAAAGGCACTTGAGTGGTTGGGTTTTATTAGAAACAAAGCTAATGGTTACACAACA
               *******.**** **************** ***********:** *******: *  *
```

```
IGHV7-3*02        GAGTACAGTGCATCTGTGAAGGGTCGGTTCACCATCTCCAGAGATAATTCCCAAAGCATC
37-/-#9           GAGTACAGTGCATCTGTGAAGGGTCGGTTCACCATCTCCAGAGATAATTCCCAAAGCATC
29+/+#2           GAGTACAGTGCATCTGTGAAGGGTCGGTTCACCATCTCCAGAGATAATTCCCAAAGCATC
64-/+#7           GAGTACAGTGCATCTGTGAAGGGTCGGTTCACCATCTCCAGAGATAATTCCCAAAGCATC
64-/+#6           GAGTATAGTGCATCTATGAAGGGTCGGATCACCATCTCCAGAGATAATTCCCAAAGTATT
79-/+#5           GAGTACAGTGCATCTGTGAAGGGTCGGTTCACCATCTCCAGAGATAATTCCCAAAGCATC
EPS/BCL2_39_1     GAGTACAGTGCATCTGTGAAGGGTCGGTTCACCATCTCCAGAGATAATTCCCAAAGCATC
EPS/BCL2_14_3     GAGTACAGTGCATCTGTGAAGGGTCGGTTCACCATCTCCAGAGATAATTCCCAAAGCATC
                  ***** *********.***********:**************************** **

IGHV7-3*02        CTCTATCTTCAAATGAACACCCTGAGAGCTGAGGACAGTGCCACTTATTACTGT
37-/-#9           CTCTTTCTTCACTTGAATACCCTGAGAGCTGAGGACAGTGCCACTTATTATTGT
29+/+#2           CTCTATCTTCAAATGAACACCCTGAGAGCTGAGGACAGTGCCACTTATTACTGT
64-/+#7           CTCTATCTTCAAGTGAACACCCTGAGAGCTGAGGACAGTGCCACTTATTACTGT
64-/+#6           TTCTATCTTCAAATGAACACCCTGAGAGCTGAGGACAGTGCCACTTATTACTGT
79-/+#5           CTCTATCTTCAAATGAACACCCTGAGAGCTGAGGACAGTGCCACTTATTACTAT
EPS/BCL2_39_1     CTCTATCTTCAAATGAACACCCTGAGAGCTGAGGACAGTGCCACTTATTACTGT
EPS/BCL2_14_3     CTCTATCTTCAAATGAACACACTGAGAGCTGAGGACAGTGCCACTTATTACTGT
                   ***:******. **** ******************************** *.*
```

	SSP	%	SHM	%	IMGT
29+/+#2	6	2	0	0	98
64-/+#7	5	1.7	2	0.7	97.6
64-/+#6	5	1.7	13	4.4	93.9
79-/+#5	3	1	2	0.7	98.3
37-/-#9	3	1	14	4.8	94.2

G

```
IGHV4-1*02F       GAGGTGAAGCTTCTCGAGTCTGGAGGTGGCCTGGTGCAGCCTGGAGGATCCCTGAAACTC
14-/-#8           CAAGTTAAGCTGCAGCAGTCTGGAGGTGGCCTGGTGCAGCCTGGAGGATCCCTGAAACTC
37-/-#4           CAAGTTCAGCTGCAGGAGTCAGGAGGTGGCCTGGTGCAGCCTGGAGGATCCCTGAAACTC
EPS/BCL2_21_4     CAAGTTAAGCTGGAGCAGTCTGGAGGTGGCCTGGTGCAGCCTGGAGGATCCCTGAAACTC
EPS/BCL2_79_16    CAGGTCAAGCTGCAGGAGTCAGGAGGTGGCCTGGTGCAGCCTGGAGGATCCCTAAAACTC
                   *.** .****   : ****:********************************.******

IGHV4-1*02F       TCCTGTGCAGCCTCAGGATTCGATTTTAGTAGATACTGGATGAGTTGGGTCCGGCAGGCT
14-/-#8           TCCTGTGCAGCCTCAGGATTCGATTTTAGTAGATACTGGATGAGTTGGGTCCGGCAGGCT
37-/-#4           TCCTGTGCAGCCTCAGGATTCGATTTTAGTAAAGACTGGATGAGTTGGGTCCGGCAGGCT
EPS/BCL2_21_4     TCCTGTGCAGCCTCAGGATTCGATTTTAGTAAAGACTGGATGAGTTGGGTCCGGCAGGCT
EPS/BCL2_79_16    TCCTGTGAAGTCTCAGGATTCGATTTTAGTAAAGACTGGATGAATTGGGTCCGGCAGTCT
                  *******.** ********************.* *********.************* **

IGHV4-1*02F       CCAGGGAAAGGGCTAGAATGGATTGGAGAAATTAATCCAGATAGCAGTACGATAAACTAT
14-/-#8           CCAGGGAAAGGGCTAGAATGGATTGGAGAAATTAATCCAGATAGCAGTACGATAAACTAT
37-/-#4           CCAGGGAAAGGGCTAGAATGGATTGGAGAAATTAATCCAGATAGCAGTACGATAAACTAT
EPS/BCL2_21_4     CCAGGGAAAGGGCTAGAATGGATTGGAGAAATTAATCCAGATAGCAGTACGATAAACTAT
EPS/BCL2_79_16    CCAGGAAAAGGACTAGAATGGATTGGAGAAATTAATCCAGATAGCAGTACGATAAACTAT
                  *****.*****.************************************************

IGHV4-1*02F       ACGCCATCTCTAAAGGATAAATTCATCATCTCCAGAGACAACGCCAAAAATACGCTGTAC
14-/-#8           ACGCCATCTCTAAAGGATAAATTCATCATCTCCAGAGACAACGCCAAAAATACGCTGTAC
37-/-#4           GCACCATCTCTAAAGGATAAATTCATCATCTCCAGAGAGAACGCCAAAAATACGCTGTAC
EPS/BCL2_21_4     GCACCATCTCTAAAGGATAAATTCATCATCTCCAGAGAGAACGCCAAAAATACGCTGTAC
EPS/BCL2_79_16    GCACCATTTCTAAGGGATAAATTCATCATCTCCAGAGAGAATGCCAAAAATACGCTGTTC
                  .*.**** *****.************************ ** ****************:*
```

```
IGHV4-1*02F        CTGCAAATGAGCAAAGTGAGATCTGAGGACACAGCCCTTTATTACTGT
14-/-#8            CTGCAAATGAGCAAAGTGAGATCTGAGGACACAGCCCTTTATTACTGT
37-/-#4            CTGCAAATGAGCAAAGTGAGATCTGAGGACACAGCCCTTTATTACTGT
EPS/BCL2_21_4      CTGCAAATGAGCAAAGTGAGATCTGAGGACACAGCCCTTTATTACTGT
EPS/BCL2_79_16     CTGCAGATGACCGAAGTGAGATCTGAGGACACAGCCCTTTATTATTGT
                   *****.**** *.******************************* ***
```

	SSP	%	SHM	%	IMGT
14-/-#8	6	2.1	1	0.35	97.6
37-/-#4	11	3.9	2	0.7	95.5

H

```
IGHV3-6*01      GATGTACAGCTTCAGGAGTCAGGACCTGGCCTCGTGAAACCTTCTCAGTCTCTGTCTCTC
EPS/BCL2_11     NANGTTAAGCTGCAGGAGTCAGGACCTGGCCTCGTGAAACCTTCTCAGACTCTGTCTCTC
EPS/BCL2_25     NANGTGAAGCTGCAGCAGTCTGGACCTGGCCTCGTGAAACCTTCTCAGACTCTGTCTCTC
71-/-#3         CAAGTTAAGCTGGAGGAGTCTGGACCTGGCCTCGTGAAACCTTCTCAGACTCTGTCTCTC
                 * **  .****   ** ****:***************************:***********

IGHV3-6*01      ACCTGCTCTGTCACTGGCTACTCCATCACCAGTGGTTATTACTGGAACTGGATCCGGCAG
EPS/BCL2_11     ACCTGCTCTGTCACTGGCTACTCCATCACCAGTGGTTATACCTGGCACTGGATCAGGCAG
EPS/BCL2_25     ACCTGCTCTGTCACTGGCTACTCCATCACCAGTGGTTATTACTGGCACTGGATCAGGCAG
71-/-#3         ACCTGCTCTGTCACTGGCTACTCCATCACCAGTGGTTATTACTGGCACTGGATCAGGCAG
                ***************************************:.****.********.*****

IGHV3-6*01      TTTCCAGGAAACAAACTGGAATGGATGGGCTACATAAGCTACGATGGTAGCAATAACTAC
EPS/BCL2_11     TTTCCAGGAAACAAACTGGAGTGGATGGGATACATAAACTACAGTGGTGGCACTAACTAC
EPS/BCL2_25     TTTCCAGGAAAAAAACTGGAGTGGATGGGATACATAAGCTACAGTGGTAGCACTAACTAT
71-/-#3         TTTCCAGGAAACAAACTGGAGTGGATGGGATACATAAGCTACAGTGGTAGCACTAACTAC
                ***********.********.********.*******.****..****.***.****** 

IGHV3-6*01      AACCCATCTCTCAAAAATCGAATCTCCATCACTCGTGACACATCTAAGAACCAGTTTTTC
EPS/BCL2_11     AACCCATCTCTCAAAAATCGAATCTCCATCACTCATGACACATCTAAGAACCAGTTCTTC
EPS/BCL2_25     AATCCATCTCTCAAAAATCGAATCTCTATCACTCATGACACATCTAAGAACCAGTTCTTC
71-/-#3         AACCCATCTCTCAAAAATCGAATCTCCATCACTCATGACACATCTAAGAGCCAGTTCTTC
                ** *********************** *******.**************.****** ***

IGHV3-6*01      CTGAAGTTGAATTCTGTGACTACTGAGGACACAGCCACATATTACTGT
EPS/BCL2_11     CTGAAGTTGAATTCTGTGACTACTGAGGACACGGCCACATATTATTGT
EPS/BCL2_25     CTGAAGTTGAATTCTGTGACTACTGAGGACACAGCCACATATTATTGT
71-/-#3         CTGAAGTTGAATTCTGTGACTACTGAGGACACAGCCACATATTATTGT
                ********************************.*********** ***
```

	SSP	%	SHM	%	IMGT
71-/-#3	12	4.2	7	2.4	93.4

Supplementary Figure 4. Comparative Clustal W analysis of the IGHV sequences from B cell clones producing identical HCDR3 sequences. **A**. Analysis of the IGHV region (IGHV14-2*01 F gene) from the *Traf2*DNx*BCL2*-tg$^{+/+}$ CLL/SLL clones encoding an HDCR3 region with the sequence GRDDGYYYAMDY. **B**. A similar analysis for the IGHV region (IGHV1-74*04 F gene) from clones encoding an HDCR3 region with the sequence ASGYDYAMDY. **C**. Analysis of the IGHV region (IGHV11-2*02 gene) from the *Traf2*DNx*BCL2*-tg$^{-/-}$ and *Traf2*DNx*BCL2*-tg$^{+/+}$ encoding the HCDR3 MRYSSYDYDWYFDV. **D**. Analysis of the IGHV region (IGHV6-6*02 F) from *Traf2*DNx*BCL2*-tg$^{+/-}$ and *Traf2*DNx*BCL2*-tg$^{+/+}$ encoding the HCDR3 TSWDVNY. Highlighted in blue are the nucleotides differing from the other sequences

A

GRDDGYYYAMDY (IGHV14-2*01 F)

```
65+/+#8         CAAGTTCAGCTGGAGGAGTCTGGGGCAGAGCTTGTGAAGCCAGGGGCCTCAGTCAAGTTG
72+/+#1         CCGGTTAAGCTGCAGGAGTCTGGGGCAGAGCTTGTGAAGCCAGGGGCCTCAGTCAAGTTG
13+/+#1         CAAGTGAAGCTGGAGGAGTCAGGGGCAGAGCTTGTGAAGCCAGGGGCCTCAGTCAAGTTG
                *..** .***** *******:***************************************

65+/+#8         TCCTGCACAGCTTCTGGCTTCAACATTAAAGACTACTATATGCACTGGGTGAAGCAGAGG
72+/+#1         TCCTGCACAGCTTCTGGCTTCAACATTAAAGACTACTATATGCACTGGGTGAAGCAGAGG
13+/+#1         TCCTGCACAGCTTCTGGCTTCAACATTAAAGACTACTATATGCACTGGGTGAAGCAGAGG
                ************************************************************

65+/+#8         CCTGAACAGGGCCTGGAGTGGATTGGAAGGATTGATCCTGAGGATGGTGAAACTAAATAT
72+/+#1         CCTGAACAGGGCCTGGAGTGGATTGGAAGGATTGATCCTGAGGATGGTGAAACTAAATAT
13+/+#1         CCTGAACAGGGCCTGGAGTGGATTGGAAGGATTGATCCTGAGGATGGTGAAACTAAATAT
                ************************************************************

65+/+#8         GCCCCGAAGTTCCAGGGCAAGGCCACTATAACAGCAGACACATCATCCAACACAGCCTAC
72+/+#1         GCCCCGAAGTTCCAGGGCAAGGCCACTATAACAGCAGACTCATCATCCAACACAGCCTAC
13+/+#1         GCCCCGAAGTTCCAGGGCAAGGCCACTATAACAGCAGACACATCATCCAACACAGCCTAC
                ***************************************:********************

65+/+#8         CTACAGCTCAGAAGCCTGACATCTGAGGACACTGCCATCTATTACTGTGGGAGA
72+/+#1         CTACAGCTCAGAAGCCTGACATCTGAGGACACTGCCATCTATTACTGTGGGAGA
13+/+#1         CTACAGCTCAGAAGCCTGACATCTGAGGACACTGCCATCTATTACTGTGGGAGA
                ******************************************************
```

B

ASGYDYAMDY (IGHV1-74*04 F)

```
50+/+#1         CAAGTTCAGCTGCAGCAGTCTGGGCCTCAGCTGGTTAGGCCTGGGGCTTCAGTGAAGATA
72F1+/+#1       CAAGTTCAGCTGCAGGAGTCTGGGCCTCAGCTGGTTAGGCCTGGGGCTTCAGTGAAGATA
55+/+#1         CAAGTCAAGCTGCAGCAGTCTGGGCCTCAGCTGGTTAGGCCTGGGGCTTCAGTGAAGATA
                ***** .******** ********************************************

50+/+#1         TCCTGCAAGGCTTCTGGTTACTCATTCACCAGCTACTGGATGCACTGGGTGAAGCAGAGG
72F1+/+#1       TCCTGCAAGGCTTCTGGTTACTCATTCACCAGCTACTGGATGCACTGGGTGAAGCAGAGG
55+/+#1         TCCTGCAAGGCTTCTGGTTACTCATTCACCAGCTACTGGATGCACTGGGTGAAGCAGAGG
                ************************************************************

50+/+#1         CCTGGACAAGGTCTTGAGTGGATTGGCATGATTGATCCTTCCGATAGTGAAACTAGGTTA
72F1+/+#1       CCTGGACAAGGTCTTGAGTGGATTGGCATGATTGATCCTTCCGATAGTGAAACTAGGTTA
55+/+#1         CCTGGACAAGGTCTTGAGTGGATTGGCATGATTGATCCTTCCGATAGTGAAACTAGGTTA
                ************************************************************
```

```
50+/+#1        AATCAGAAGTTCAAGGACAAGGCCACATTGACTGTAGACGAATCCTCCAGCACAGCCTAC
72F1+/+#1      AATCAGAAGTTCAGGGACAAGGCCACATTGACTGTAGACAAATCCTCCAGCACAGCCTAC
55+/+#1        AATCAGAAGTTCAAGGACAAGGCCACATTGACTGTAGACAAATCCTCCAGCACAGCCTAC
               *************.*************************.********************

50+/+#1        ATGCAACTCAGCAGCCCGACATCTGAGGACTCTGCGGTCTATTACTGTGCAA
72F1+/+#1      ATGCAACTCAGCAGCCCGACATCTGAGGACTCTGCGGTCTATTACTGTGCAA
55+/+#1        ATGCAACTCAGCAGCCCGACATCTGAGGACTCTGCGGTCTATTACTGTGCAA
               ****************************************************
```

C

MRYSSYDYDWYFDV (IGHV11-2*02)

```
47-/-#1        --TGTGCAGCTGGTGGAGTCTGGAGGAGGCTTGGTG-AACCTGGGGGGTTACGGGGACTC
14-/-#2        TTTGTGCAGCTGGTGGAGTCTGGAGGAGGCTTGGTGCAACCTGGGGGGTCACGGGGACTC
40+/+#2        CAAGTTAAGCTGCAGCAGTCAGGAGGAGGCTTGGTGCAACCTGGGGGGTCACGGGGACTC
                 :** .***** :* ****:*************** ************ **********

47-/-#1        TCTTGTGAAGGCTCAGGGTTTACTTTTAGTGGCTTCTGGATGAGCTGGGTTCGACAGACA
14-/-#2        TCTTGTGAAGGCTCAGGGTTTACTTTTAGTGGCTTCTGGATGAGCTGGGTTCGACAGACA
40+/+#2        TCTTGTGAAGGCTCAGGGTTTACTTTTAGTGGCTTCTGGATGAGCTGGGTTCGACAGACA
               ************************************************************

47-/-#1        CCTGGGAAGACCCTGGAGTGGATTGGAGACATTAATTCTGATGGCAGTGCAATAAACTAC
14-/-#2        CCTGGGAAGACCCTGGAGTGGATTGGAGACATTAATTCTGATGGCAGTGCAATAAACTAC
40+/+#2        CCTGGGAAGACCCTGGAGTGGATTGGAGACATTAATTCTGATGGCAGTGCAATAAACTAC
               ************************************************************

47-/-#1        GCACCATCCATAAAGGATCGATTCACTATCTTCAGAGACAATGACAAGAGCACCCTGTAC
14-/-#2        GCACCATCCATAAAGGATCGATTCACTATCTTCAGAGACAATGACAAGAGCACCCTGTAC
40+/+#2        GCACCATCCATAAAGGATCGATTCACTATCTTCAGAGACAATGACAAGAGCACCCTGTAC
               ************************************************************

47-/-#1        CTGCAGATGAGCAATGTGCGATCTGAGGACACAGCCACGTATTTCTGTATGAGATA
14-/-#2        CTGCAGATGAGCAATGTGCGATCTGAGGACACAGCCACGTATTTCTGTATGAGATA
40+/+#2        CTGCAGATGAGCAATGTGCGATCTGAGGACACAGCCACGTATTTCTGTATGAGATA
               ********************************************************
```

D

TSWDVNY (IGHV6-6*02 F)

```
58+/-#6        GAGGTCAAGCTGGAGGAGTCAGGAGGAGGCTTGGTGCAACCTGGAGGATCCATGAAACTC
72F1+/+#2      GAGGTTAAGCTGGAGGAGTCTGGAGGAGGCTTGGTGCAACCTGGAGGATCCATGAAACTC
24+/-#1        CAAGTTAAGCTGCAGGAGTCAGGAGGAGGCTTGGTGCAACCTGGAGGATCCATGAAACTC
                *.** ****** *******:***************************************

58+/-#6        TCCTGTGTTGCCTCTGGATTCACTTTCAGTAACTACTGGATGAACTGGGACCGCCAGTCT
72F1+/+#2      TCCTGTGTTGCCTCTGGATTCACTTTCAGTAACTACTGGATGAACTGGGTCCGCCAGTCT
24+/-#1        TCCTGTGTTGCCTCTGGATTCACTTTCAGTAACTACTGGATGAACTGGGTCCGCCAGTCT
               *************************************************:**********

58+/-#6        CCAGAGAAGGGGCTTGAGTGGGTTGCTGAAATTAGATTGAAATCTAATAATTATGCAACA
72F1+/+#2      CCAGAGAAGGGGCTTGAGTGGGTTGCTGAAATTAGATTGAAATCTAATAATTATGCAACA
24+/-#1        CCAGAGAAGGGGCTTGAATGGGTTGCTGAAATTAGATTGAAATCTAATAATTATGCAACA
               *****************.******************************************
```

```
58+/-#6         CATTATGCGGAGTCTGTGAAAGGGAGGTTCACCATCTCAAGAGATGATTCCAAAAGTAGT
72F1+/+#2       CATTATGCGGAGTCTGTGAAAGGGAGGTTCACCATCTCAAGAGATGATTCCAAAAGTAGT
24+/-#1         CATTATGCGGAGTCTGTGAAAGGGAGGTTCACCATCTCAAGAGATGATTCCAAAAGTAGT
                ************************************************************

58+/-#6         GTCTACCTGCAAATGAACAACTTAAGAGCTGAAGACACTGGCATTTATTACTGTACCAG
72F1+/+#2       GTCTACCTGCAAATGAACAACTTAAGAGCTGAAGACACTGGCATTTATTACTGTACCAG
24+/-#1         GTCTACCTGCAAATGAACAACTTAAGAGCTGAAGACACTGGCATTTATTACTGTACCAG
                ***********************************************************
```
